# HSPA6 Promotes Ferroptosis in Triple-Negative Breast Cancer by Rewiring Lipid Metabolism to Potentiate Membrane Lipid Peroxidation

**DOI:** 10.7150/ijbs.129745

**Published:** 2026-04-08

**Authors:** Lin-Yue Hai, Zhi-Hao Yu, Wen-Bo Liu, Yuan-Yuan Zhang, Yue Xu, Shan Cheng, Hao-Ran Yue, Zhang-Yin Guo, Shi-Jie Yan, Rui Sun, Xiao-Feng Liu, Bo-Wen Liu, Xin Wang, Xu-Chen Cao, Yue Yu

**Affiliations:** 1 The First Department of Breast Cancer, Tianjin Medical University Cancer Institute and Hospital, National Clinical Research Center for Cancer, Tianjin 300060, China.; 2 Key Laboratory of Cancer Prevention and Therapy, Tianjin 300060, China.; 3 Tianjin's Clinical Research Center for Cancer, Tianjin 300060, China.; 4 Key Laboratory of Breast Cancer Prevention and Therapy, Tianjin Medical University, Ministry of Education, Tianjin 300060, China.; 5 Shanghai Pudong Hospital, Fudan University, Shanghai, People's Republic of China, Shanghai 201200, China.

**Keywords:** ferroptosis, HSPA6, lipid metabolism, Lands cycle, ROS

## Abstract

Given the lack of effective targeted therapeutic options for triple-negative breast cancer (TNBC), there is an imperative demand for innovative treatment approaches, with ferroptosis standing out as a promising direction. This study identifies HSPA6 as a key ferroptosis sensitizer in TNBC. Mechanistically, HSPA6 binds to NF-κB p65, inhibits its nuclear translocation and Ser468 phosphorylation, thereby suppressing transcription of the lipogenic enzyme FASN and downregulating phospholipid-remodeling enzymes LPCAT1/cPLA2. This dual inhibition enriches membrane phospholipids with polyunsaturated fatty acids, heightening peroxidation susceptibility and triggering ferroptosis. Concurrently, HSPA6-mediated suppression of lipogenesis depletes palmitate, thereby attenuating ANKIB1 palmitoylation and inhibiting its E3 ligase activity. This impairs K48-linked ubiquitination and degradation of HSPA6, forming a stabilizing positive feedback loop. Our study uncovers a HSPA6-p65-FASN-ANKIB1 axis linking lipid metabolism to ferroptosis, offering a novel TNBC therapeutic target.

## Introduction

Breast cancer is the most frequently diagnosed cancer and the leading cause of cancer-related death among women worldwide, followed by lung and colorectal cancer in both incidence and mortality^1^. Triple-negative breast cancer (TNBC), defined by the lack of expression of estrogen receptor (ER), progesterone receptor (PR), and human epidermal growth factor receptor 2 (HER2)^2^, is a particularly aggressive subtype. This receptor-negative profile renders TNBC intrinsically resistant to endocrine and HER2-directed therapies, confining standard care primarily to chemotherapy and radiotherapy and complicating clinical management. Accounting for 15-20% of all breast cancer cases, TNBC is linked with a disproportionately poor prognosis; approximately one-third of patients develop distant metastases, ultimately leading to disease-related death^3^. Consequently, identifying novel TNBC biomarkers is critical for formulating personalized therapeutic strategies, assessing treatment response, and predicting prognosis, thereby addressing a significant unmet medical need^4^. TNBC is characterized by elevated levels of reactive oxygen species (ROS), which confers susceptibility to ferroptosis^5^. Therefore, targeting key regulators of ferroptosis represents a potential therapeutic strategy^6^.

Ferroptosis, an intracellular iron-dependent form of regulated cell death distinct from apoptosis, necrosis, and autophagic cell death, was initially defined by Stockwell and colleagues in 2012^7^, ^8^. Mechanistically, it is driven by the peroxidation of polyunsaturated fatty acid-containing phospholipids (PUFA-PLs) within cellular membranes. This lethal lipid peroxidation is exacerbated by dysregulated iron metabolism and specific mitochondrial pathways, culminating in the accumulation of phospholipid hydroperoxides (PLOOHs) that directly induce cell death^9^. Cellular defense against ferroptosis primarily relies on the canonical glutathione peroxidase 4 (GPX4)/glutathione(GSH) axis and GPX4-independent pathways, such as the ferroptosis suppressor protein-1 (FSP1)/coenzyme Q10(CoQ10) system. Critically, ferroptosis plays pivotal roles in multiple facets of cancer biology, including tumor initiation, progression, metastasis, cancer stemness maintenance, and the development of therapy resistance^10^. Inducing ferroptosis in tumor cells represents a promising therapeutic strategy for cancer treatment.

Altered lipid metabolism is a recognized metabolic hallmark of breast cancer^11^. Enhanced lipid metabolism drives cancer progression and survival by upregulating fatty acid synthesis and promoting lipid droplet accumulation, which is achieved through either increased exogenous lipid uptake or *de novo* lipogenesis^12^. This metabolic adaptation not only meets the elevated energy demands of cancer cells but also helps mitigate treatment-induced reactive oxygen species (ROS)^13^. Fatty acids (FAs), as fundamental lipid components, exert biological functions through not only their cellular abundance but also structural characteristics, specifically carbon chain length and degree of unsaturation. Of note, the stoichiometric balance among saturated (SFAs), monounsaturated (MUFAs), and polyunsaturated fatty acids (PUFAs) governs cellular homeostasis and membrane functionality^14^. Notably, elevated PUFA incorporation into phospholipid membranes relative to MUFAs increases membrane susceptibility to peroxidation, thereby driving ferroptosis sensitivity^15^. Mounting evidence suggests that cell-autonomous enzymatic defense systems actively detoxify lipid peroxides, constituting a key mechanism of ferroptosis suppression. Targeting aberrant lipid desaturation, a process involved in tumorigenesis and progression, significantly suppresses tumor growth, metastasis, and relapse in preclinical models^16^. The Lands cycle represents the principal pathway for the remodeling of phospholipids (PLs), a process dedicated to the exchange and modification of their FA composition^17^. The Lands cycle enables cells to restrict lipid peroxidation and ferroptosis by preferentially incorporating less susceptible fatty acids over PUFAs into membrane phospholipids^18^. Consequently, genes and metabolic pathways involved in lipid peroxide repair, antioxidant defense, and PUFA metabolism collectively dictate a cell's propensity for ferroptosis.

Heat shock 70-kDa protein 6 (HSPA6) is located at human chromosomal locus 1q23.3 and encodes a conserved 70-kDa molecular chaperone^19^. It is constitutively expressed in multiple tissues and displays inducible expression under proteotoxic stressors, including heat shock, oxidative stress, cancer, and toxic insults^20^. Accumulating evidence indicates that HSPs are involved in the pathophysiological process of ferroptosis. Although HSPA6 shares > 90% coding sequence identity with the adjacent non-coding pseudogene HSPA7^21^, its specific role and mechanistic contributions to cancer pathogenesis have remained elusive since its discovery three decades ago. Existing evidence suggests that HSPA6 may function as a tumor suppressor in TNBC^22^, ^23^, its relationship with ferroptosis remains unknown.

This study establishes HSPA6 as a critical ferroptosis promoter in TNBC pathogenesis. Mechanistically, HSPA6 represents a novel upstream regulator controlling p65 nuclear import via direct interaction and modulation of its Ser468 phosphorylation/importin-binding state. HSPA6 orchestrates lipid metabolic reprogramming by coordinating *de novo* lipogenesis and the lands cycle, two key pathways that mediate ferroptotic susceptibility. Furthermore, an HSPA6-p65-FASN-ANKIB1 signaling axis that drives tumorigenesis was identified as a therapeutic target for TNBC.

## Materials and Methods

### Bioinformatics analysis

RNA sequencing data and clinical data were retrieved from The Cancer Genome Atlas (TCGA) (https://portal.gdc.cancer.gov/), the Genotype-Tissue Expression (GTEx) (https://gtexportal.org/), and the Gene Expression Omnibus (GEO) (https://www.ncbi.nlm.nih.gov/geo/). The TCGA and GEO databases are open access and publicly available, and data access policies and guidelines were followed during this study.

### Reagents

Antibodies against HSPA6 (13616-1-AP, Proteintech, 1:2000 for WB, and 1:100 for IHC, RRID:AB_10896881), β-actin (66009-1-Ig, Proteintech, RRID:AB_2492035), FTH1 (YM8481, Immunoway, RRID:AB_10564673), GPX4 (YM8430, Immunoway, RRID:AB_10896855), FASN (10624-2-AP, Proteintech, RRID:AB_1642031), N-cadherin (13116, Cell Signaling Technology, RRID:AB_2314843), Survivin (2808, Cell Signaling Technology, RRID:AB_2864590), Vimentin (5741, Cell Signaling Technology, RRID:AB_2492283), LPCAT1 (YN5768, Immunoway, RRID:AB_10644931), cPLA2 (YT1084, Immunoway, RRID:AB_2799425), and IgG rabbit (ZB2301, ZSGB-BIO) were used. Small molecule inhibitors included TVB3166 (HY-120394, MedchemExpress), Erastin (HY-15763, MedchemExpress), and Ferrostatin-1 (HY-100579, MedchemExpress) were used.

### Drug sensitivity analysis

Drug sensitivity profiles, half-maximal inhibitory concentration (IC50) values, and corresponding mRNA expression data were obtained from 265 compounds screened across 860 cell lines from the Genomics of Drug Sensitivity in Cancer (GDSC) database and compounds screened in 1,001 cell lines from the Cancer Therapeutics Response Portal (CTRP) database. Next, these datasets were integrated to assess associations between compound IC50 values and gene mRNA expression levels using Pearson correlation analysis. All P-values underwent false discovery rate (FDR) correction for multiple hypothesis testing.

### Tissue samples

A total of 40 matched pairs of primary TNBC and adjacent normal tissues were collected from patients who underwent surgical resection at Tianjin Medical University Cancer Institute and Hospital (TMUCIH; Tianjin, China), with pathology confirming diagnosis. Among these, samples from 10 patients were reserved fresh for subsequent quantitative real-time PCR (qPCR) and Western blot analyses. All protocols were approved by the Ethical Committee of Tianjin Medical University Cancer Institute and Hospital. Informed and written consent was obtained from all participating patients.

### Immunohistochemistry

Tissue specimens were formalin-fixed, paraffin-embedded, and sectioned at a thickness of 5 μm. Then, the slides were incubated at 60 °C for 2 hours, followed by deparaffinization in xylene, rehydration through a graded ethanol series, and antigen retrieval using citrate buffer (pH 6.0). Endogenous peroxidase activity was quenched with 3% H₂O₂ for 15 minutes at room temperature. Non-specific binding was blocked with 3% BSA for 1 hour at 37°C prior to incubation with primary antibodies overnight at 4°C. After washing, the sections were incubated with horseradish peroxidase (HRP)-conjugated anti-rabbit/mouse secondary antibodies (PV9000, ZSGB-Bio) for 60 minutes at 37°C. Immunoreactivity was visualized using 3,3'-diaminobenzidine (DAB) substrate (ZLI-9017, ZSGB-Bio) for 5-10 minutes at room temperature. Afterward, the sections were counterstained with Mayer's hematoxylin, dehydrated through a graded alcohol series, cleared in xylene, and mounted with synthetic resin. Lastly, images were acquired using a brightfield microscope (Axio Imager M2, Zeiss) equipped with a 20× objective. All sections were captured and estimated by three independent pathologists using a dual-parameter scoring system: positive staining intensity scores (no staining, 0; weak staining, 1; moderate staining, 2; strong staining, 3) and the expression extent scores (< 25%, 1; 25-50%, 2; 50-75%, 3; > 75%, 4).

### Western blot

Cells were lysed in RIPA buffer (Solarbio, #R0020) containing protease and phosphatase inhibitors (Solarbio, #P0100). Protein extracts (10-30 µg) were separated by SDS-PAGE and transferred onto PVDF membranes. Membranes were blocked with 5% non-fat dry milk (Solarbio, #D8340) in TBST (Tris-buffered saline with 0.5% Tween-20) and then incubated with primary antibodies overnight at 4 °C. After incubation with appropriate HRP-conjugated secondary antibodies the protein signals were detected using enhanced chemiluminescence (ECL).

### Reverse transcriptase PCR (RT-PCR) and quantitative real-time PCR (qPCR)

Total RNA was isolated using the RNA Extraction Kit (TAKARA, #9767) and reverse-transcribed into cDNA with the TransScript cDNA Synthesis system (TransGen, #AT311-02). Quantitative RT-PCR was performed with One-Step RT-qPCR SuperMix (TransGen, #AQ211-01) following manufacturer's protocols. Gene expression levels were normalized to β-actin and quantified via the 2^-ΔΔ^Ct method. Primer sequences are provided in Table S1.

### Cell culture

Human breast cancer cell lines MDA-MB-231 (RRID:CVCL_0062), BT-549 (RRID:CVCL_1092), CAL-51 (RRID:CVCL_1110), the non-tumorigenic mammary epithelial line MCF-10A (RRID: CVCL_0598), and HEK 293T (RRID: CVCL_0063) cells were sourced from the Cell Bank of the Chinese Academy of Sciences (Shanghai, China) between May and July 2023 and maintained under standard culture conditions as validated in prior studies^24^. Mycoplasma contamination was excluded on arrival and every three months thereafter using a PCR-based assay.

### Plasmid transfection

Plasmid transfections were performed using Lipofectamine 3000 (Invitrogen, #L3000015) following manufacturer's protocol. shRNAs targeting the HSPA6 coding sequence were designed as follows: shHSPA6#1: 5'-GAGAGCGCAACGTGCTCATTT-3', shHSPA6#2: 5'-CCATTGACGCTGGTGTCTTTG-3', shHSPA6#3: 5'-ATCCAGAGGAACGCCACTATC-3'. A non-targeting scrambled shRNA served as the negative control: shNC: 5'-CAACAAGATGAAGAGCACCAA-3'.

### Lentivirus packaging and infection

Lentiviral particles for HSPA6 overexpression and knockdown were generated by co-transfecting 293T cells with engineered transfer plasmids and the Lenti-Pac HIV Packaging Kit (iGeneBio, #LV001). After 48 h, viral supernatants were harvested, passed through 0.45 μm filters, and concentrated with Lenti-X Concentrator (Takara, #631232). Breast cancer cells were transduced with viral particles in medium containing 8 μg/ml hexadimethrine bromide (Polybrene; Solarbio, #H8761). Stable cell lines were selected under 1 μg/ml puromycin (Merck, #P9620) for 14 days and validated by western blot.

### CCK-8 and colony formation assays

Cell viability was assessed using a Cell Counting Kit-8 (CCK-8; Beyotime, C0038). Briefly, the cells were seeded in the 96-well plate at a density of 1,000 cells per well in 100 µl of DMEM supplemented with 10% FBS. At designated time points, 10 µl of CCK-8 solution was added to each well, followed by incubation at 37 °C for 1 h under 5% CO₂. Absorbance was measured at 450 nm using a microplate reader, with a reference wavelength of 650 nm for background subtraction.

Colony formation assays were performed by seeding TNBC cells (1,000 cells/well) in six-well plates. After 14 days of culture, colonies were fixed with 4% paraformaldehyde (PFA) for 15 min at room temperature, stained with 0.1% crystal violet (Solarbio, C8470) for 15 min, and air-dried prior to quantification.

### Wound healing and transwell assays

TNBC cells were plated to full confluency on a 6-well dish, subjected to scratching, and then cultured under serum-free conditions for an additional 24 h. Images were captured at 0 and 24 h to document changes in wound width. TNBC cells (80,000 cells/well) were seeded into the upper chamber of 24-well Transwell plates with 8 μm-pore size (Corning, USA) without Matrigel coating, while the lower chamber was filled with DMEM containing 10% FBS as a chemoattractant. After incubating for 8-24 h, non-migrating cells on the upper side of the chamber were removed by scrubbing, and migrating cells on the lower side were fixed with 4% paraformaldehyde and stained with crystal violet.

### Co-immunoprecipitation (Co-IP) assay

Cell lysates were prepared using IP lysis buffer (Beyotime, P0013) and clarified by centrifugation at 12,000 × g for 15 min at 4 °C. Thereafter, the clarified lysates were incubated with primary antibodies overnight at 4 °C under constant rotation to allow the formation of immune complexes. Protein A/G magnetic beads (Thermo Fisher, 88802) were pre-washed with IP lysis buffer and then incubated with immune complexes for 1 h at 4 °C with rotation. They were subsequently washed five times with IP lysis buffer and once with ultrapure water. Bound proteins were eluted by resuspending beads in 2× SDS loading buffer and boiling at 95 °C for 10 min. Eluates were subjected to Western blotting and mass spectrometry analyses.

### Xenograft

Female NOD/SCID/IL2 receptor γ-null (NSG) mice (5-6 weeks old) were acquired from SPF (Beijing) biotechnology Co. Ltd. and housed in the Animal Research Center's specific pathogen-free facility at TMUCIH. All experimental protocols and husbandry for animal studies were authorized by the ethics committee of the TMUCIH (Approval No: 2024063). All animal experiments were approved by the Animal Ethics Committee and were conducted in accordance with the animal welfare guidelines.

For subcutaneous xenograft models, 1 × 10^7^ stable expressing cells (MDA-MB-231-vector/HSPA6) suspended in 150μL PBS/Matrigel mixture (4:1) were implanted into the right flanks of each group (n = 5).

For metastatic models, 5 × 10^5^ stable expressing cells (MDA-MB-231-vector/HSPA6) were intravenously injected via the tail vein of each group (n = 5). Metastatic clone formation was assessed by bioluminescence imaging every 7 days using *in vivo* imaging systems.

For Erastin treatment models, 1×10^7^ stable expressing cells (MDA-MB-231-vector/HSPA6) suspended in 150 μL PBS/Matrigel mixture (4:1) were implanted into the right flanks of each group (n = 5). Once the tumors had reached a volume of approximately 20mm³, each group received intratumoral injections (100 μL) of erastin (10 mg/kg) every 4 days for a total of 8 doses.

For FASNi treatment models, mice were randomly allocated into 4 groups (n = 5), then implanted with 1×10^7^ stable expressing cells. Once the tumors had reached a volume of approximately 20mm³, 3 groups received daily administered via oral gavage of TVB-3166 (30 mg/kg/100 μL/mice), dissolved in 0.2% DMSO-PBS. The fourth group was treated with oral administration of 0.2% DMSO-PBS.

Pre-established exclusion criteria were: (i) failure of tumor engraftment (volume < 50 mm³ by day 10), (ii) death unrelated to treatment (e.g., gavage error), or (iii) tumor burden exceeding the humane endpoint (volume > 2000 mm³ or ulceration).

Tumor dimensions were measured every 5 days using digital calipers, with volume calculated as V = (L×W^2^)/2. For xenograft studies, mice were euthanized when xenografts reached 1000 mm^3^ or at the end of the study, whichever came first. The tumor tissues were embedded in paraffin and stained for hematoxylin and eosin, IHC staining.

### Oil red O staining

Cells were treated with either 0.2 µM FASNi or 0.2% DMSO (vehicle control) for 48 h. Following treatment, cells were washed twice with PBS, fixed with 4% formaldehyde for 60 min at room temperature, and rinsed twice with PBS. Lipid droplets were stained by sequential incubation with 60% isopropanol (5 min) and 0.3% Oil Red O (C0157S, Beyotime) in isopropanol (20 min, room temperature).

### Iron, MDA, NADPH and GSH level measurement

Following treatment, 2 × 10^6^ TNBC cells were harvested and resuspended in PBS for further analysis. Iron levels were determined using the Iron Assay Kit (S1066S, Beyotime), while MDA levels were assessed with the MDA Assay Kit (S0131S, Beyotime), NADPH levels were assessed with the NADPH Assay Kit (S0180S, Beyotime), and GSH levels were measured using the GSH Assay Kit (S0053, Beyotime). Assays were conducted according to the manufacturer's protocols. Absorbance was measured at 593 nm (iron level), 532 nm (MDA level), 450 nm (NADPH level), and 412 nm (GSH level). Standard curves were generated to determine their concentrations.

### Mitochondrial membrane potential (MMP) measurement

JC-1 was detected using a mitochondrial membrane potential detection kit (C2003S, Beyotime). Briefly, after incubating with the JC-1 staining work solution for 20 min at 37 °C, the cells were trypsinized, resuspended in PBS containing 2% FBS, and analyzed on a CytoFLEX LX cytometer (Beckman Coulter) equipped with 488-nm and 561-nm lasers. Data were processed using employed FlowJo v10.8 with fluorescence compensation applied. The red fluorescent aggregate indicates a healthy mitochondrion with normal membrane potential, whereas the green fluorescent monomer indicates loss of MMP.

### ROS and lipid peroxidation measurement

Intracellular ROS levels were quantified using 2',7'-dichlorodihydrofluorescein diacetate (DCFH-DA; Beyotime, S0033M). Stock solutions (10 mM in DMSO) were diluted to working concentrations of 10 μM (DCFH-DA, 1:1000) in serum-free medium. Cells were loaded with probes at 37 °C under 5% CO₂ for 25 min in the dark. The fluorescent probe DCFH-DA diffuses into cells, where it is enzymatically hydrolyzed to DCFH and subsequently oxidized by intracellular ROS to generate fluorescent DCF. The fluorescence intensity of DCF thus serves as an indicator of cellular ROS levels. After washing twice with PBS, live-cell fluorescence imaging was performed using an Eclipse Ti2 inverted microscope (Nikon) with FITC (Ex/Em 488/525 nm) channels. For flow cytometry, cells were trypsinized, resuspended in PBS containing 2% FBS, and analyzed on a CytoFLEX LX cytometer (Beckman Coulter) equipped with 488 nm lasers. Data were processed using employed FlowJo v10.8 with fluorescence compensation applied.

### Dual-luciferase reporter assay

HEK293T cells were seeded in 6-well plates and transfected upon reaching 70-80% confluency using Lipofectamine 3000 (Thermo Fisher, L3000001) according to the manufacturer's protocol. Each well was transfected with 2 μg of experimental plasmid, 10 ng pRL-SV40 of Renilla luciferase internal control plasmid (Promega, E2231), and pcDNA3.1 empty vector to maintain 4 μg total DNA. After 48 h, cells were lysed in 300 μL Passive Lysis Buffer (Promega, E1941) containing protease inhibitors (Roche, 11873580001). Lysates were cleared by centrifugation (12,000 × g, 10 min, 4 °C). Firefly and Renilla luciferase activities were sequentially quantified using the Dual-Luciferase Reporter Assay System (Promega, E1910) on a GloMax Multi+ Luminometer (Promega) with 10-second integration per read. Firefly signals were normalized to Renilla activity and expressed as relative luminescence units (RLU).

### Chromatin immunoprecipitation assay (ChIP)

Cells were cross-linked with 1% formaldehyde for 15 min at room temperature, and the reaction was terminated by adding 125 mM glycine for 30 min. Chromatin was then fragmented into 200-500 bp pieces by sonication. The sheared chromatin was incubated with primary antibodies overnight at 4 °C. Magnetic beads (Millipore, USA) were added the next day, followed by incubation for an additional 4 h at 4 °C with gentle rotation. The bead-bound complexes were collected, washed, and eluted. After reversing cross-links at 65 °C, samples were treated with RNase A and proteinase K. DNA was purified by phenol-chloroform extraction and analyzed by quantitative real-time PCR (qPCR). The purified DNA was analyzed by PCR with primers listed in Table S1.

### Acyl-biotin exchange assay

The Acyl-Biotin Exchange (ABE) assay was performed to detect S-palmitoylation of ANKIB1 as described^25^ with modifications. Briefly, cells were lysed in RIPA buffer (Thermo Fisher, 89900) containing 50 mM N-ethylmaleimide (NEM; Sigma, E3876) and protease inhibitors (Roche, 11873580001). Following this, the lysates were incubated at 4 °C for 1 h with end-over-end mixing, followed by chloroform/methanol precipitation. ANKIB1 was immunoprecipitated overnight at 4 °C using anti-ANKIB1 antibody (Cell Signaling, 12948S; 1:100) conjugated to Protein G magnetic beads (Thermo Fisher, 10004D). Beads were washed thrice with lysis buffer and subsequently treated with 1 M hydroxylamine (HAM; pH 7.2; Sigma, 255580) or Tris-HCl control for 1 h at 25°C. Next, palmitoylation sites were labeled with 5 mM biotin-BMCC (Cayman Chemical, 10006642) in PBS/1% SDS for 2 h at 4 °C under rotation. Bead complexes were resuspended in 2× Laemmli buffer (Bio-Rad, 1610737), denatured at 95 °C for 10 min, and resolved by SDS-PAGE. Biotinylated ANKIB1 was detected by Western blot using streptavidin-HRP (Abcam, ab7403).

### Cu(I)-catalyzed azide-alkyne cycloaddition reaction

Cells were cultured on coverslips in a 12-well plate. Then, they were incubated with 20 µM arachidonic acid alkyne (Cayman Chemical, 10538) for 6 h. After fixation with 3% paraformaldehyde in PBS for 15 min and permeabilization with 0.25% Triton X100 in PBS for 15 min, cells were washed with 1% BSA in PBS. The Click-iT reaction cocktail was prepared according to the manufacturer's instructions. After staining nuclei with DAPI, cells were rewashed and mounted on a slide using Fluoromount-G medium.

### Protein purification and GST binding assay

DNA fragment corresponding to HSPA6 was cloned into the pGEX-4T-1 vector. The GST and GST-HSPA6 proteins were expressed in the BL21 strain. Cells were harvested and disrupted by sonication in PBS supplemented with a complete protease inhibitor cocktail. The clear lysate was applied onto a gravity flow column containing glutathione resin. Purified HSPA6 proteins were then mixed with glutathione resin, and the binding reaction was incubated for 2 h at 4 ℃ Precipitates were washed extensively with lysis buffer. Proteins bound to glutathione beads were eluted with 10 mM reduced glutathione in 50 mM Tris-HCl, and detected by immunoblotting with indicated antibodies.

### Transmission electron microscope

Following the respective treatment, cells were harvested and fixed overnight at 4 °C in 2.5% glutaraldehyde. Typically, after the initial fixation, cells were rinsed three times with 0.1 M PBS, followed by a secondary fixation in osmium tetroxide for 1 h. The samples were then subjected to dehydration through a graded ethanol series and subsequently embedded in acrylic resin. Ultrathin sections were stained using uranyl acetate and lead citrate before imaging. Transmission electron micrographs were acquired using an FEI Tecnai Spirit microscope operating at 120 kV.

### MS analysis

For qualitative protein analysis, HEK293T cells were transiently transfected with a plasmid encoding Flag-tagged HSPA6. Cells were subsequently lysed, and Flag-HSPA6-containing complexes were immunoprecipitated using anti-Flag agarose beads. The immunoprecipitated complexes were resolved by SDS-PAGE and visualized with Coomassie Blue staining. Protein bands of interest were excised, subjected to in-gel trypsin digestion, and vacuum-dried. Protein composition and post-translational modifications were then analyzed by mass spectrometry (MS) following established protocols^26^. For cell proteome analysis, protein was extracted by lysing the sample, and protein concentrations were quantified using the BCA method. Equal amounts of proteins were subjected to denaturation and reductive alkylation treatment, followed by enzymatic digestion using trypsin at 37 ℃ for 2 h. Afterward, the supernatant was concentrated using a C18 column at 45 ℃ for use, and resuspended for the subsequent analysis. One microgram of protein per sample was subjected to LC-MS/MS analysis according to the standard protocols^27^.

### Untargeted metabolomics

The samples were placed in the EP tubes and resuspended with prechilled 80% methanol by well vortex. Then the samples were melted on ice and whirled for 30 s. After the sonification for 6 min, they were centrifuged at 5,000 rpm, 4 °C for 1 min. The supernatant was freeze-dried and dissolved with 10% methanol. Finally, the solution was injected into the LC-MS/MS system analysis^28^.

### Electrophoretic mobility shift assay (EMSA)

Tsingke (Beijing, China) provided biotin-labeled RNA oligonucleotides. The Chemiluminescent EMSA Kit (Beyotime, China, GS009) was utilized to perform the RNA EMSA assay, following the guidelines provided by the manufacturer. For EMSA, 5 µg of cell nuclear protein was incubated with 1 nmol biotin-labeled probe in the binding buffer for 20 min at room temperature. For competitive EMSA, 100 nmol of unlabeled probes were added into the reaction mixture 20 min prior to the addition of a constant amount of the labeled positive probe. For super shift assays, proteins were preincubated with antibodies at a temperature of 37 °C for a duration of 20 min before labeled probes were added. The reaction mixtures were resolved on 6.0% non-denaturing polyacrylamide gels and transferred to nylon membranes (Beyotime, China). UV crosslinking was used to bind the RNA oligomers to the membrane, and the chemiluminescent imaging system was employed to detect the labeled probes.

### Statistical analysis

All experiments were performed in triplicate with at least three biologically independent replicates. Data were expressed as mean ± standard deviation (SD) unless otherwise specified. Statistical analyses were conducted using GraphPad Prism 8.0 (GraphPad Software). Pairwise comparisons were carried out using two-tailed unpaired Student's t-tests with Welch's correction for unequal variances. Multi-group comparisons were conducted using one-way or two-way ANOVA, followed by Tukey's honestly significant difference post hoc test. Statistical significance thresholds were defined as *P < 0.05, **P < 0.01, and ***P < 0.001; ns denotes non-significance (P ≥ 0.05). All data met assumptions of normality (Shapiro-Wilk test) and homogeneity of variance (Brown-Forsythe test).

## Results

### HSPA6 is a crucial suppressor protein associated with ferroptosis in TNBC

The critical involvement of ferroptosis in tumor progression and therapy resistance positions it as an emerging therapeutic paradigm in oncology^29^, ^30^. To identify potential ferroptosis-related genes playing vital roles in breast tumorigenesis, the transcriptional levels of 180 ferroptosis-related genes were analyzed from the GeneCards database and published literature, leading to the identification of 49 differentially expressed ferroptosis-related genes in BRCA. These differentially expressed genes (DEGs) were subsequently visualized using volcano and heat maps, as illustrated in Figure S1A and Figure S1B. The corresponding log2-fold change and adjusted p-value for the 49 DEGs are detailed in Table S2. Moreover, univariate Cox regression analysis identified 11 candidate prognostic genes with p < 0.05, as presented in Figure S1C. Subsequently, LASSO (Least Absolute Shrinkage and Selection Operator) Cox regression analysis was applied to identify and retain the most prognostically relevant Ferroptosis-Related Genes (FRGs) while mitigating the effects of multicollinearity among covariates (Figure S1D), leading to the identification of nine ferroptosis-related genes. The gene set was optimized using regression coefficient calculations (Figure S1E and Figure S1F). Next, BRCA patients were stratified into G1 (n = 484) and G2 (n = 485) groups based on the median ferroptosis group score. Kaplan-Meier analysis with log-rank testing demonstrated significantly shorter overall survival (OS) in the G2 group compared to the G1 group (Figure S1G). Furthermore, the association between group stratification (G1/G2) and key clinicopathological characteristics was investigated. Interestingly, the G2 group was significantly associated with older age, advanced T stage, and lymph node metastasis (Figure S1H). Finally, a prognostic nomogram was constructed incorporating the ferroptosis group score, Pathologic T stage, Pathologic N stage, Pathologic M stage, age, and predicted 1-, 3-, and 5-year survival probabilities (Figure S1I). TNBC poses significant clinical challenges, highlighting the need for novel therapeutic strategies. A deeper understanding of the regulatory mechanisms governing ferroptosis may offer valuable insights^29^. Prior studies have indicated that TNBC cells exhibit elevated iron and lipid abundance, rendering them particularly susceptible to ferroptosis induction, a phenomenon that holds significant therapeutic and prognostic implications^29^. To evaluate the clinical relevance of the developed ferroptosis signature, its prognostic utility was assessed in independent TNBC cohorts sourced from the GEO database. Specifically, datasets GSE58812 (n = 107) and GSE651216 (n = 55), comprising TNBC samples with comprehensive clinical annotation, served as validation cohorts. Consistent with findings in the training cohort, Kaplan-Meier analysis revealed significantly reduced survival probability in the G2 subgroup compared to G1 within the GSE58812 dataset (Figure S1J) and GSE65216 datasets (Figure S1J). To systematically identify ferroptosis-regulating genes and their mechanistic roles in TNBC, cross-cohort comparative analysis of differentially expressed genes (DEGs) among ferroptosis subgroups across the TCGA, GSE58812, and GSE65216 datasets was performed, identifying consistent dysregulation of HSPA6 expression, primarily upregulated in group G1, suggesting its potential role in ferroptosis modulation in TNBC (Figure S1K).

**Figure 1:**
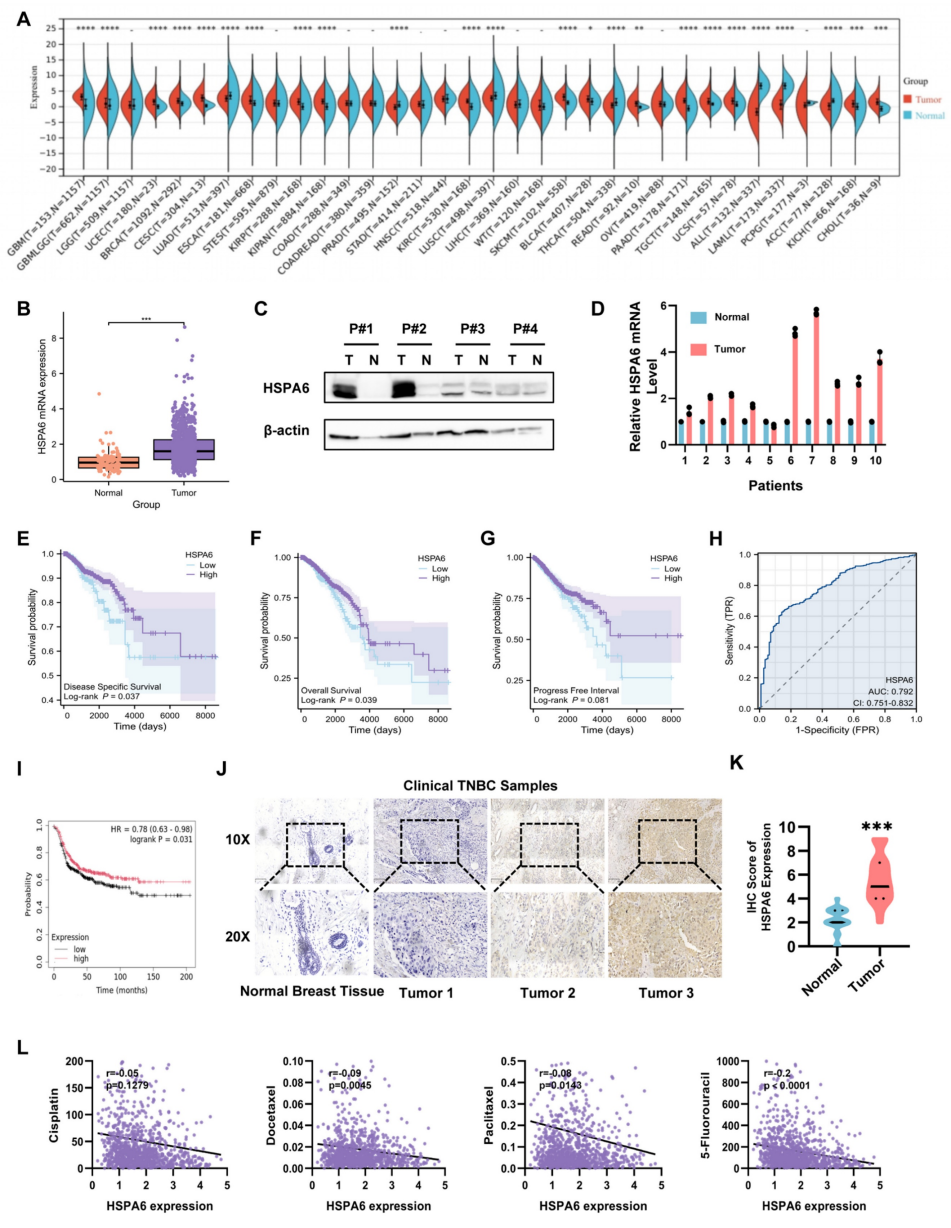
** HSPA6 is a crucial suppressor protein associated with ferroptosis in TNBC. A)** The expression of HSPA6 in tumor and adjacent-healthy tissues from TCGA database.** B)** The expression of HSPA6 in breast cancer and adjacent-healthy tissues from TCGA database (n_Tumor_=1085, n_Normal_=291). **C)** The protein expression of HSPA6 in breast cancer and adjacent-healthy tissues in patients with TNBC determined by Western blot. **D)** The HSPA6 mRNA expression levels in breast cancer and adjacent-healthy tissues in patients with TNBC determined by qPCR. **E-G)** Kaplan-Meier analysis of overall survival (**E**), disease-specific survival (**F**), and progression-free survival (**G**) in breast cancer patients with different HSPA6 expression level (n_Low_=449, n_High_=638). **H)** The HSPA6 expression level as an independent prognostic factor in patients with BRCA by ROC analysis. **I)** Kaplan-Meier analysis of overall survival in TNBC patients with different HSPA6 expression level from TCGA database (n_Low_=449, n_High_=638).** J and K)** The expression of HSPA6 TNBC tissues and adjacent-healthy tissues determined by immunochemistry. n = 40, Scale bars, 100 μm. **L)** The correlation between the HSPA6 expression and IC_50_ values for cisplatin, Docetaxel, Paclitaxel, and 5-Flu. (Data are presented as mean ± standard of error (SD) of three independent experiments. Statistical significance was determined using ANOVA with post-hoc Tukey multiple comparison, * P < 0.05, ** P < 0.01, *** P < 0.001, ns.: not significant.)

Under conditions of cellular stress or in tumor cells, the expression of heat shock proteins typically undergoes marked upregulation to facilitate cellular adaptation to adverse environments and maintain cell viability. Similarly, analysis of public transcriptomic databases (TIMER2.0 and GEPIA) revealed significant upregulation of HSPA6 mRNA across multiple cancer types, including BRCA (Figure 1A and Figure 1B). Western blot analysis of four paired TNBC and adjacent healthy tissue specimens confirmed elevated HSPA6 protein expression levels in tumor tissues (Figure 1C). In line with these findings, RT-qPCR quantification demonstrated a significant increase in HSPA6 transcript levels in 10 additional paired TNBC clinical samples compared to matched normal controls (Figure 1D). Analysis of GEO datasets uncovered a significant upregulation of HSPA6 mRNA in low-grade malignancies and improved survival outcomes in patients with TNBC (Figure S1L). To further investigate the clinical relevance of HSPA6 in cancer progression, its expression in the TCGA-BRCA cohort was analyzed. As anticipated, patients with low HSPA6 expression levels exhibited significantly poorer survival outcomes compared to the high-expression group, including shorter overall survival, progression-free interval and disease-specific survival (Figure 1E-G). ROC analysis demonstrated robust prognostic value for HSPA6 expression (Figure 1H). It is worth noting that a significant association between HSPA6 expression and prognosis was also observed in the TCGA TNBC cohort (Figure 1I). It is posited that a potential association exists between HSPA6 and TNBC cell survival. Mechanistically, elevated HSPA6 protein levels in TNBC compared to healthy breast tissues were validated by IHC (Figure 1J and Figure 1K), consistent with findings from the Human Protein Atlas (HPA) database (Figure S1M). Furthermore, HSPA6 expression was negatively correlated with IC50 values for cisplatin, Docetaxel, Paclitaxel, and 5-Flu (Figure 1L), suggesting its role in modulating chemosensitivity. Collectively, these findings position HSPA6 as a key regulator of TNBC progression and a potential prognostic biomarker.

### HSPA6 suppresses growth and metastasis of TNBC both *in vitro* and *in vivo*

**Figure 2:**
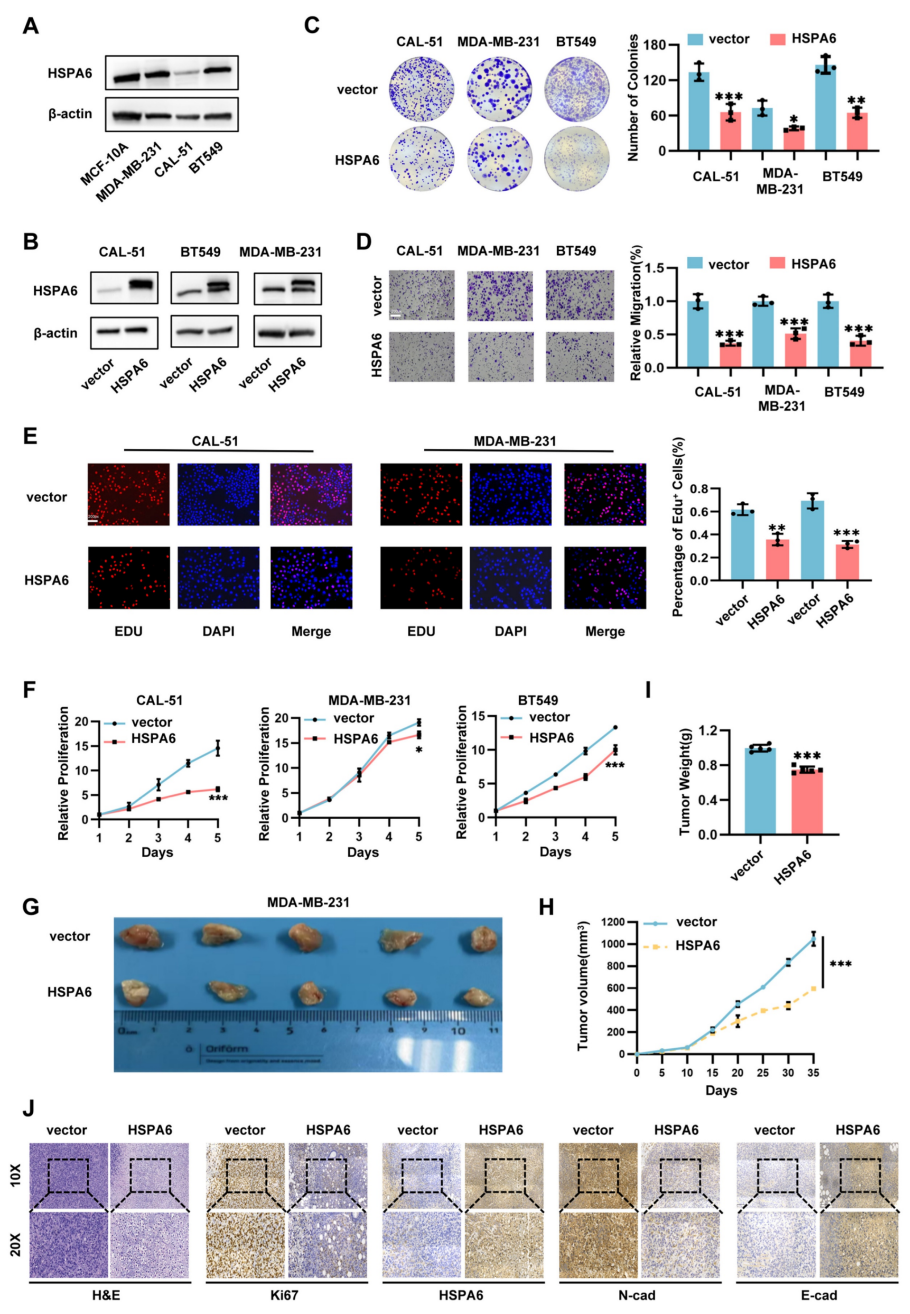
** HSPA6 suppressed growth and metastasis of TNBC *in vitro* and *in vivo*. A)** The expression level of HSPA6 in MCF-10A and TNBC cell lines determined by Western blot. **B)** The expression of HSPA6 in HSPA6-overexpressing as well as the control cells verified by Western blot. **C)** Clonogenic assays to examine the effects of HSPA6 overexpression as well as the control cells on TNBC cell proliferation. **D)** Transwell assays to evaluate the effects of HSPA6 overexpression on TNBC cell invasion, scale bar, 200μm. **E)** The proliferation of CAL-51 and MDA-MB-231 cells was evaluated using EdU assays, scale bar, 50μm. **F)** The viability of HSPA6-overexpression and control cells determined by CCK-8 assays. **G)** Representative xenograft images from nude mice subcutaneously implanted with HSPA6-overexpressing MDA-MB-231 or control cells. **H)** Tumor volume of xenografts.** I)** Tumor weight of xenografts. **J)** The expression of HSPA6, Ki67, E-cadherin, and N-cadherin in xenografts of each group were assessed by immunochemistry. Scale bar, 100 μm. (Data are presented as mean ± standard of error (SD) of three independent experiments. Statistical significance was determined using ANOVA with post-hoc Tukey multiple comparison, * P < 0.05, ** P < 0.01, *** P < 0.001, ns.: not significant.)

While the functional role of HSPA6 in TNBC remains to be elucidated, quantitative analysis of endogenous HSPA6 expression across TNBC cell lines revealed marked heterogeneity. Specifically, MDA-MB-231 and BT549 cells exhibited significantly elevated HSPA6 levels, whereas CAL-51 demonstrated near-undetectable expression (Figure 2A). This differential expression pattern suggests context-dependent functional roles in TNBC pathogenesis. To delineate the functional contribution of HSPA6 to TNBC pathogenesis, we employed a lentiviral-based gain-of-function approach was employed to overexpress HSPA6 in MDA-MB-231, CAL-51, and BT549 cell lines (Figure 2B). In parallel, three independent shRNAs targeting distinct HSPA6 sequences were designed for loss-of-function studies in MDA-MB-231 and BT549 cells, with shRNA#1 and shRNA#2 selected for subsequent experiments (Figure S2A). Functional assessment of HSPA6 in TNBC progression revealed that enforced HSPA6 overexpression significantly impaired clonogenic capacity, whereas HSPA6 knockdown enhanced proliferative potential across the tested cell lines (Figure 2C, Figure S2B). Given the established role of epithelial-mesenchymal transition (EMT) in driving metastasis^31^, ^32^, the impact of HSPA6 on EMT markers was explored. Western blot analysis demonstrated that HSPA6-overexpressing MDA-MB-231 cells exhibited downregulation of mesenchymal markers N-cadherin and Vimentin expression (Figure S2D). This coordinated induction of EMT-associated factors implies that HSPA6 may attenuate mesenchymal programming in TNBC. Functional interrogation of HSPA6 in TNBC metastasis revealed that genetic depletion enhanced migratory capacity in wound healing and Transwell assays, while its overexpression suppressed cell motility (Figure 2D, Figure S2C, Figure S2F).

To assess systemic metastatic potential, firefly luciferase-tagged MDA-MB-231 cells (MDA-MB-231-Luc) stably expressing HSPA6 were established via lentiviral transduction. Quantitative bioluminescence imaging at 6 weeks post-intravenous inoculation demonstrated a significant reduction in metastatic burden in HSPA6-overexpressing cohorts compared to vector controls (Figure S2G). Proliferation analyses further indicated that HSPA6 overexpression impaired cell growth, as evidenced by reduced CCK-8 absorbance and diminished EdU-positive cell fractions (Figure 2F, Figure S2E, Figure S2H). Next, we used animal experiments to investigate the role of HSPA6 on TNBC growth *in vivo*. These results of subcutaneous xenograft tumor models in nude mice showed that tumors with HSPA6 overexpression were significantly smaller, grew more slowly, and had reduced weights compared to the control group (Figure 2H-I). Immunohistochemical (IHC) staining showed that E-cad levels were significantly increased, while Ki67 and N-cad levels were decreased in HSPA6 overexpression tumors compared to control tumors. Collectively, these data establish HSPA6 as a multifunctional regulator suppressing TNBC progression through coordinated inhibition of proliferation, migration, and metastatic colonization.

### HSPA6 induces ferroptosis of TNBC cells both *in vitro* and *in vivo*

**Figure 3:**
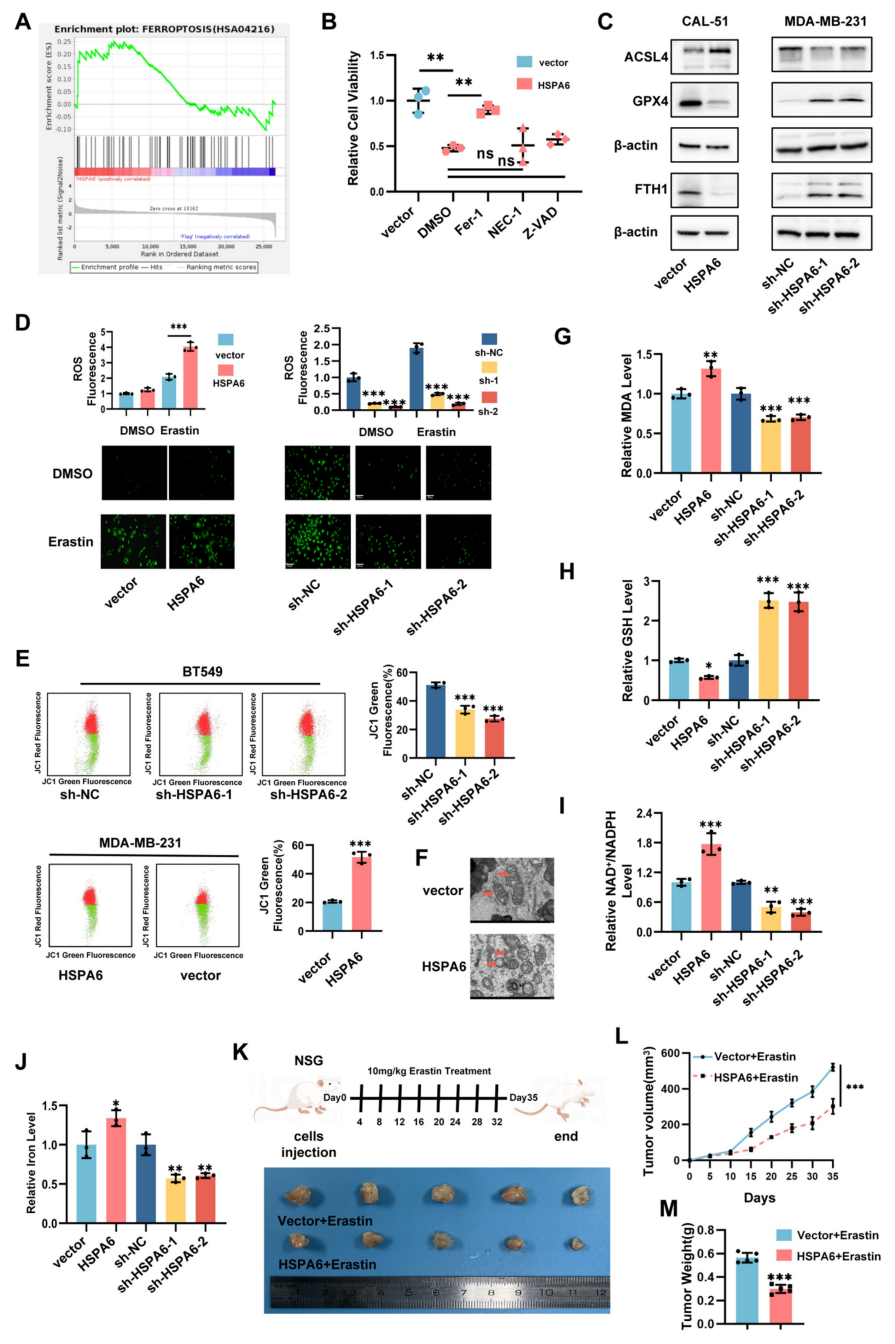
** HSPA6 facilitates ferroptosis and attenuates TNBC tumorigenesis.** GSEA of altered HSPA6 expression and ferroptosis pathway. **B)** The viability of HSPA6-overexpressing TNBC cells treated with pan-caspase inhibitor Z-VAD, NEC-1, or ferroptosis inhibitor Fer-1 for 48 hours, as well as the control cells determined by CCK-8 assay. **C)** The expression levels of ferroptosis-related biomarkers GPX4, FTH1, and ACSL4 in HSPA6-overexpressing or HSPA6-depleted TNBC cells, as well as control cells determined by Western blot. **D)** The Lipid ROS level in HSPA6-overexpressing or HSPA6-depleted TNBC cells, as well as control cells was detected by fluorescence microscope, scale bar, 200μm. **E)** Mitochondrial membrane potential was measured using the JC-1 probe. The distribution of JC-1 aggregates (PE channel) and monomers (FITC channel) in HSPA6-overexpressing or HSPA6-depleted TNBC cells, as well as control cells was determined by flow cytometry. **F)** The Intracellular MDA level were measured by corresponding assay kits in HSPA6-overexpressing or HSPA6-depleted TNBC cells, as well as control cells. **G)** The Intracellular GSH level were measured by corresponding assay kits in HSPA6-overexpressing or HSPA6-depleted TNBC cells, as well as control cells. **H)** The Intracellular NAD^+^/NADPH level were measured by corresponding assay kits in HSPA6-overexpressing or HSPA6-depleted TNBC cells, as well as control cells. **I)** The Intracellular Iron content were measured by corresponding assay kits in HSPA6-overexpressing or HSPA6-depleted TNBC cells, as well as control cells. **J)** HSPA6 caused morphological changes in cellular mitochondria, including smaller size, reduced cristae, and even membrane rupture (transmission electron microscopy). Scale bars, 100 μm. **K)** Schematic diagram showing the experimental design for xenograft model. Representative xenograft images from nude mice subcutaneously implanted with HSPA6-overexpressing MDA-MB-231 or control cells treated by Erastin (10mg/kg) or DMSO-PBS. **L and M)** Growth curves (L) and tumor weight (M) of the subcutaneous xenografts in each group was measured. (Data are presented as mean ± standard of error (SD) of three independent experiments. Statistical significance was determined using ANOVA with post-hoc Tukey multiple comparison, * P < 0.05, ** P < 0.01, *** P < 0.001, ns.: not significant.)

To delineate the mechanistic basis of HSPA6-mediated tumor suppression in TNBC, RNA sequencing was performed on HSPA6-overexpressing TNBC cell lines. Gene set enrichment analysis (GSEA) identified significant enrichment of differentially expressed genes in core ferroptosis pathways (Figure 3A). To delineate the mode of cell death mediated by HSPA6, vector and HSPA6-overexpressing TNBC cells were treated with the pan-caspase inhibitor Z-VAD-FMK, the necroptosis inhibitor Necrostatin-1, and the ferroptosis inhibitor Ferrostatin-1 for 48 hours. Quantitative cell viability assays demonstrated that Ferrostatin-1, but not Z-VAD-FMK or Necrostatin-1, significantly reversed the HSPA6-induced growth suppression (Figure 3B), establishing ferroptosis as the primary executor of HSPA6-mediated cell death in TNBC. Meanwhile, the results of the CCK-8 assay demonstrated that HSPA6 overexpression markedly promoted RSL3-induced cell death (Figure S3A). Overall, these findings establish ferroptosis as the principal mechanism underlying HSPA6-mediated suppression of TNBC progression. To delineate the molecular basis of this effect, the expression of core ferroptosis regulators in HSPA6-modulated TNBC cells was detected. qPCR and Western blot analysis revealed that enforced HSPA6 overexpression significantly downregulated the expression of ferroptosis inhibitors FTH1 and GPX4, while concurrently upregulating that of pro-ferroptotic ACSL4 at the protein and mRNA levels. Conversely, HSPA6 depletion elevated FTH1 and GPX4 expression, accompanied by reduced levels of ACSL4 (Figure 3C, Figure S3B and Figure S3C), demonstrating a bidirectional regulatory effect of HSPA6 on the ferroptotic machinery. The effects of HSPA6 expression on ferroptosis were further investigated. Quantitative analysis revealed that enforced HSPA6 overexpression significantly elevated cellular ROS levels in TNBC cells (Figure 3D). Given the critical role of mitochondrial integrity in redox homeostasis, the impact of HSPA6 on mitochondrial function was examined. Given that mitochondria are the primary source of intracellular ROS, we then examined mitochondrial membrane integrity by measuring the mitochondrial membrane potential (MMP) and directly visualizing mitochondrial structure using transmission electron microscopy (TEM). Our results demonstrated that MMP increased significantly after HSPA6 depletion, while MMP decreased after HSPA6 overexpression (Figure 3E). Moreover, we observed the disappearance of mitochondrial cristae after HSPA6 overexpression (Figure 3F). These morphological alterations were consistent with characteristic features of ferroptosis. Concomitant measurements of oxidative stress parameters demonstrated increased malondialdehyde (MDA) levels (Figure 3G), a decreased glutathione levels (Figure 3H, and Figure S3D), alongside an elevated NADP^+^/NADPH ratio (Figure 3I), and accumulation of the labile iron pool (Figure 3J). Functionally, HSPA6 overexpression attenuated Fer-1-induced growth and clonogenic potential, whereas these effects were effectively reversed by Erastin (Figure S3E). These coordinated alterations conjointly indicate that HSPA6 disrupts mitochondrial redox homeostasis, establishing a permissive environment for ferroptosis execution.

To further elucidate the role of HSPA6 in ferroptosis and breast cancer, xenograft tumor models were constructed by subcutaneously injecting MDA-MB-231 cells with lentivirus-mediated HSPA6 overexpression, as well as an intraperitoneal injection of Erastin or DMSO-PBS (Figure 3K). Quantitative analysis at the endpoint (Day 35) demonstrated that tumor volume (Figure 3L) and tumor weight (Figure 3M) were significantly lower in the HSPA6 overexpression group compared to vector controls. Notably, erastin treatment synergistically enhanced tumor suppression in HSPA6-overexpressing tumors.

Immunohistochemical analysis of xenograft tissues demonstrated that HSPA6 overexpression significantly reduced the Ki-67⁺ proliferative index, downregulated the expression of FTH1 and N-cadherin, while elevating that of E-cadherin and ACSL4 (Figure S3F). Consistent with murine findings, analysis of human TNBC specimens revealed an inverse correlation between HSPA6 and FTH1 expression, and a positive correlation between HSPA6 and ACSL4 expression (Figure S3G). Immunofluorescence co-staining further corroborated ACSL4 upregulation and FTH1 suppression in HSPA6-high TNBC tissues (Figure S3H). Collectively, these orthogonal analyses establish HSPA6 as a key regulator of ferroptosis in TNBC through coordinated modulation of iron metabolism (FTH1) and lipid peroxidation (ACSL4) pathways.

### HSPA6 inhibits *de novo* lipogenesis and the Lands cycle

**Figure 4:**
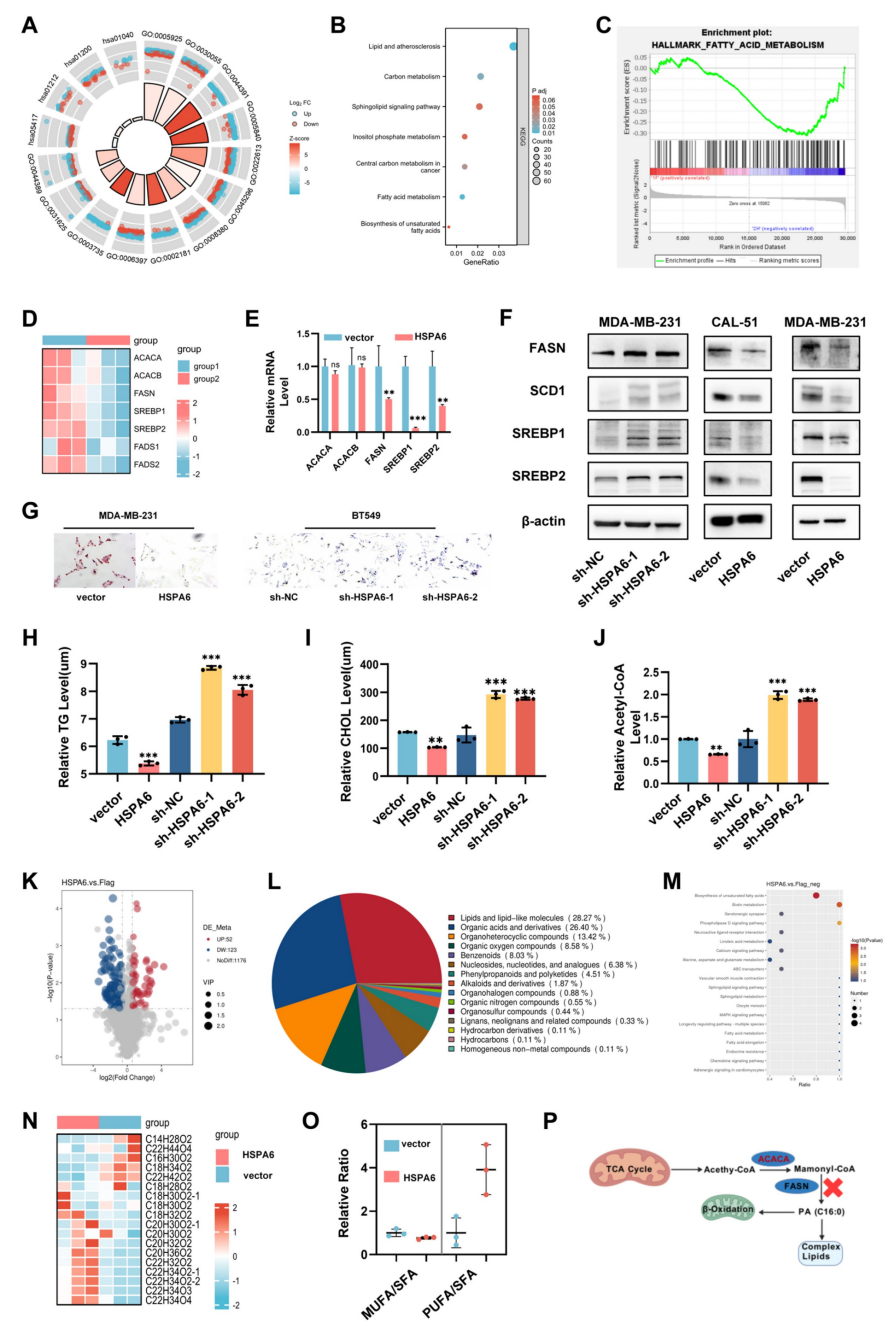
** HSPA6 inhibits *de novo* lipogenesis by decreasing FASN in TNBC. A and B)** Enrichment analysis of the differentially expressed genes by GO (A) and KEGG (B) analysis. **C)** GSEA of altered HSPA6 expression level and fatty acid metabolism pathway. **D)** The differentially expressed fatty acid metabolism-related genes in HSPA6-overexpressing MDA-MB-231 and control cells. **E and F)** The expression levels of fatty acid metabolism-related genes ACACA, ACACB, FASN, SREBP1 and SREBP2 in HSPA6-overexpressing or HSPA6-depleted TNBC cells, as well as controls determined by RT-qPCR (E) and Western blot (F). **G)** Oil Red O staining for HSPA6-overexpressing MDA-MB-231, HSPA6-depletion BT549, and control cells. **H-J)** The levels of intracellular TG (H), CHOL (I) and Acetyl-oA(J) in HSPA6-overexpressing or HSPA6-depleted TNBC cells, as well as control cells were measured. **K)** The volcano map shows significantly changed lipid species as determined by untargeted metabolomics. **L and M)** Pie charts and bubble charts are employed to visually represent the metabolic pathways that are significantly enriched with identified metabolites. **N)** HSPA6 overexpression significantly elevated PUFA-phospholipids (PUFA-PLs). **O)** SFA, PUFA, and MUFA ratio based on untargeted metabolomics between HSPA6-overexpressing and control cells. **P)** Schematic of the HSPA6/FASN axis. HSPA6 inhibits the *de novo* lipogenesis by FASN, thereby blocking the synthesis of complex lipids and ß-oxidation (FAO). (Data are presented as mean ± standard of error (SD) of three independent experiments. Statistical significance was determined using ANOVA with post-hoc Tukey multiple comparison, * P < 0.05, ** P < 0.01, *** P < 0.001, ns.: not significant.)

**Figure 5:**
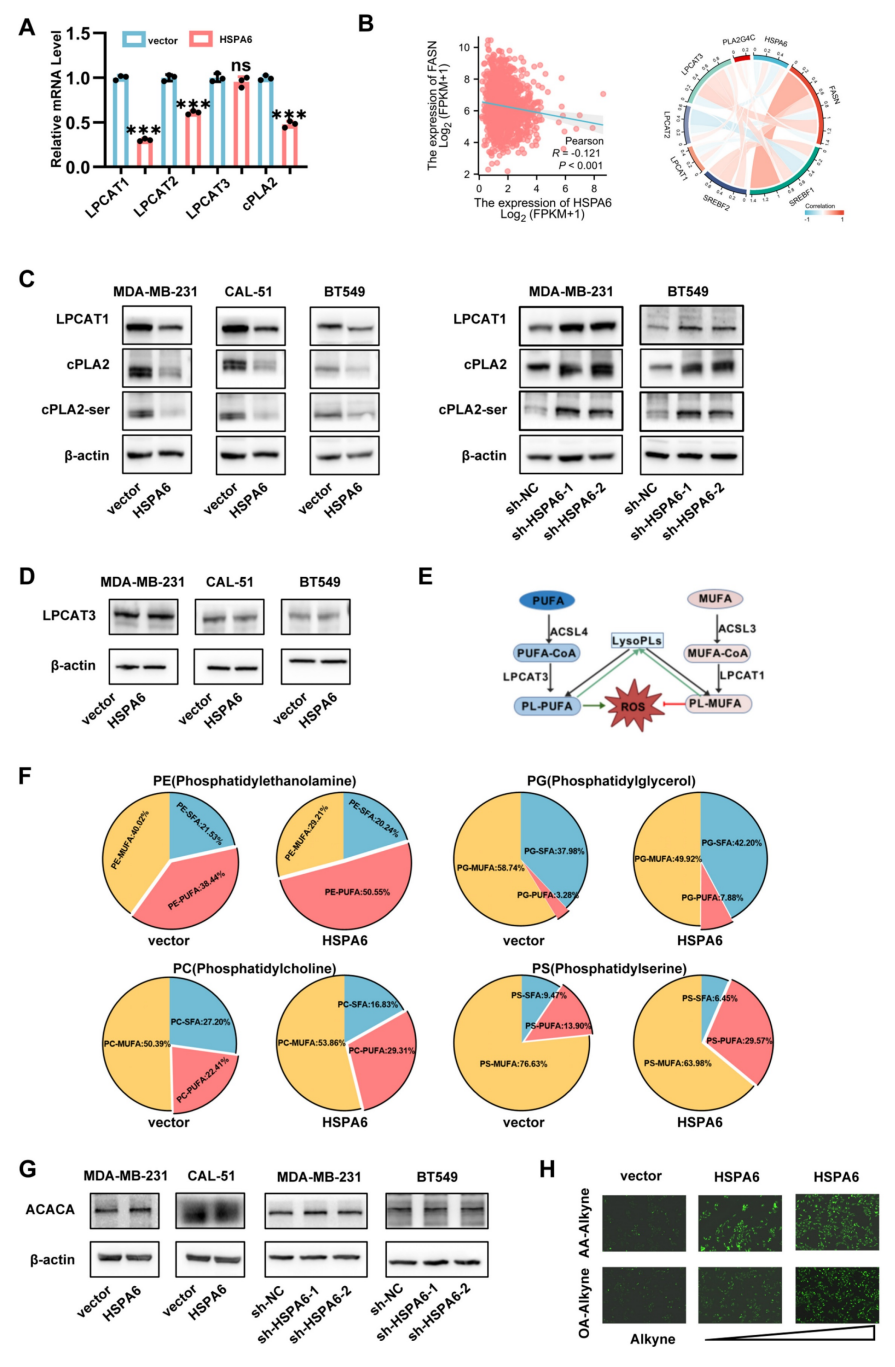
** HSPA6 decreases membrane phospholipid saturation via inhibiting the Lands cycle in TNBC.** The mRNA level of Lands cycle associated genes was performed by using RT-qPCR. **B)** Correlation between HSPA6 and Lands cycle expression in public BRCA databases. **C)** The expression levels of Lands cycle genes, including LPCAT1, cPLA2, and cPLA2-ser in HSPA6-overexpressing TNBC cells, as well as controls were detected by western blot. **D)** The expression levels of LPCAT3 in HSPA6-overexpressing TNBC cells, as well as controls were detected by western blot.** E)** Working model explaining the regulation of the Lands cycle. **F)** HSPA6 shapes the phospholipid profile in MDA-MB-231 cells.** G)** The expression levels of ACACA in HSPA6-overexpressing or HSPA6-depleted TNBC cells, as well as controls detected by western blot. **H)** Incorporation of AA and OA alkyne in the indicated cell lines treated as indicated. (Data are presented as mean ± standard of error (SD) of three independent experiments. Statistical significance was determined using ANOVA with post-hoc Tukey multiple comparison, * P < 0.05, ** P < 0.01, *** P < 0.001, ns.: not significant.)

To delineate the mechanistic basis underlying HSPA6-mediated ferroptosis sensitization, an integrated analysis of RNA sequencing data was carried out. Gene Ontology (GO) and KEGG pathway enrichment analyses revealed significant associations with lipid metabolic processes, including fatty acid metabolism, carbon metabolism, and unsaturated fatty acid biosynthesis (Figure 4A and Figure 4B). GSEA further confirmed enrichment in fatty acid metabolism pathways (Figure 4C). Given the established role of fatty acid synthase (FASN) as the rate-limiting enzyme in *de novo* lipogenesis^33^, which is frequently dysregulated in cancer^12^, ^34^, the analysis focused on lipogenic enzymes. Transcriptomic profiling demonstrated differential expression of FASN and related fatty acid synthesis components (Figure 4D and Figure 4E). Acetyl-CoA carboxylase 1 (ACACA) has been established to mediate fatty acid synthesis through *de novo* lipogenesis, and its increased activity promotes adipocyte differentiation and fat accumulation^35^. However, HSPA6 did not significantly influence the mRNA or protein expression levels of ACACA (Figure 5G). Western blot analysis revealed that HSPA6 overexpression concertedly downregulated the expression of master lipogenic regulators, namely SREBP1, FASN, SREBP2, and SCD1 (Figure 4F). Oil Red O staining further displayed that HSPA6 overexpression in TNBC cells led to a significant reduction in the number of intracellular lipid droplets (Figure 4G). Additionally, HSPA6 overexpression markedly decreased intracellular triglyceride levels, whereas HSPA6 knockdown resulted in a significant increase in the level of intracellular triglycerides (Figure 4H). Parallel reductions in occurred in cholesterol and acetyl-CoA levels were also noted (Figure 4I and Figure 4J). These findings establish HSPA6 as a master suppressor of *de novo* lipogenesis through coordinated downregulation of FASN-dependent lipogenic programming.

Given the complexity of lipid metabolism and the diversity of lipid species^36^, ^37^, untargeted metabolomics profiling was performed to characterize HSPA6-mediated lipidomic remodeling in TNBC cells. Volcano plot analysis revealed extensive metabolite dysregulation (Figure 4K), with lipids constituting 68.4% of differentially abundant metabolites (Figure 4L). Metabolic pathway enrichment analysis unveiled profound perturbation in fatty acid metabolic pathways (Figure 4M). Given that membrane PUFA enrichment, particularly over MUFAs, enhances ferroptosis susceptibility^15^, the composition of phospholipid-bound FAs was quantified. HSPA6 overexpression significantly elevated the levels of PUFA-phospholipids (PUFA-PLs) (Figure 4N). As previously described, differential fatty acid biosynthesis is of high relevance, given that various long-chain PUFAs are oxidized during ferroptosis^38^, ^39^. Thus, the PUFA:SFA ratio in phospholipids is a critical determinant of ferroptosis sensitivity. Notably, the PUFA:SFA ratio was highly enriched in HSPA6^high^ cancer cells (Figure 4O). Taken together, these results indicate that the activation of HSPA6 inhibits the effect of FASN on FA synthesis (Figure 4P).

To determine whether FASN inhibition mediates HSPA6-driven ferroptosis, TNBC cells were treated with TVB-3166, a selective FASN enzymatic inhibitor. Pharmacological FASN suppression significantly reduced cell viability (Figure S4A and Figure S4B) and abrogated HSPA6 knockdown-induced FASN upregulation (Figure S4C). Consistent with this finding, the high level of *de novo* lipogenesis in TNBC cells was largely abrogated upon pharmacological inhibition of FASN (Figure S4D-S4F). More importantly, FASN inhibition rescued ferroptotic resistance in HSPA6-depleted cells, as evidenced by attenuated lipid ROS (Figure S4G), mitochondrial membrane potential (Figure S4H), NADP^+^/NADPH level (Figure S4K), and MDA level (Figure S4L). Similarly, FASN inhibition alleviated the stimulated effects of HSPA6 downexpression on the increased levels of colonies (Figure S4I) and GSH (Figure S4J). To investigate whether FASN inhibition promotes exogenous PUFA incorporation, we utilized arachidonic acid (AA, 20:4n-6) was used as a model PUFA. Copper-catalyzed azide-alkyne cycloaddition was utilized to conjugate AA-alkyne to Alexa Fluor 488-azide^40^. Quantitative imaging revealed that baseline AA incorporation was lower in sh-HSPA6 cells compared to control cells, and FASN inhibition selectively enhanced AA uptake in these cells (Figure S4M). These observations establish FASN as a critical executor of HSPA6-mediated lipogenic suppression, through which HSPA6 governs ferroptosis susceptibility in TNBC (Figure S4N).

The Lands cycle, an intracellular phospholipid remodeling process involving phospholipase A2 (PLA2)-mediated deacylation and lysophospholipid acyltransferase (LPLAT)-catalyzed reacylation^41^, incorporates polyunsaturated acyl chains into phospholipids, with LPCAT1 serving as its key regulator. This cycle maintains membrane integrity by repairing peroxidized phospholipids. Conversely, its dysregulation drives membrane peroxidation, thereby triggering ferroptosis^42^, ^43^. Yet, the significance of the Lands cycle in the context of TNBC remains largely unexplored. RNA sequencing revealed HSPA6 overexpression suppressed the activity of enzymes implicated in Lands cycle (Figure 5A). TCGA analysis confirmed inverse correlations between the expression of HSPA6 and that of FASN, LPCAT1, and PLA2G4A (Figure 5B). At the same time, Western blot analysis validated that HSPA6 overexpression down-regulated the expression of LPCAT1, total cPLA2, and phospho-cPLA2 (Ser505) (Figure 5C). However, LPCAT3 expression was largely unaffected by HSPA6 overexpression (Figure 5A and 5D). PC are synthesized *via* the Kennedy pathway^44^. The majority of PL synthesized via the Kennedy pathway are remodeled through the Lands cycle, which consists of the de-acylation of PC and re-acylation of LysoPC^43^. The Lands cycle plays a pivotal role in supplying essential substrates that drive lipid peroxidation, thereby serving as a fundamental mechanism in the execution of ferroptosis (Figure 5E). The PUFA ratio in phospholipids is critical for ferroptosis sensitivity, in this study, HSPA6 overexpression led to a marked increase in PUFA incorporation into phospholipids (Figure 5F). These results further support our conclusions that both *de novo* FA synthesis and PL remodeling are necessary for preventing PUFA accumulation and ferroptosis in TNBC cells. In agreement with previous results in this study, HSPA6 enhanced the exogenous uptake of PUFA (Figure S4M). Since the HSPA6-mediated inhibition of *de novo* fatty acid synthesis limits raw materials for lipid peroxidation, the role of HSPA6 on the exogenous uptake of PUFA and MUFA was examined, and the results indicated that HSPA6 significantly promoted the cellular uptake of exogenous FA. Specifically, the results of the Click-iT assay demonstrated that cells overexpressing HSPA6 exhibited markedly enhanced capacity for internalizing PUFA (AA-Alkyne) compared to MUFA (OA-Alkyne) (Figure 5H). Consistently, IHC of human TNBC tissues revealed reduced FASN, LPCAT1, and cPLA2 expression levels in HSPA6-high tumors (Figure S5A). Comparable results were observed in murine xenografts (Figure S5B), while multiplex-IHC confirmed LPCAT1 downregulation in HSPA6-high human specimens (Figure S5C). These orthogonal data establish HSPA6 as a potent inhibitor of the Lands cycle, exacerbating membrane peroxidation and ferroptosis sensitivity.

### HSPA6 inhibits the translocation of p65 protein into the nucleus in an importin-dependent manner

**Figure 6:**
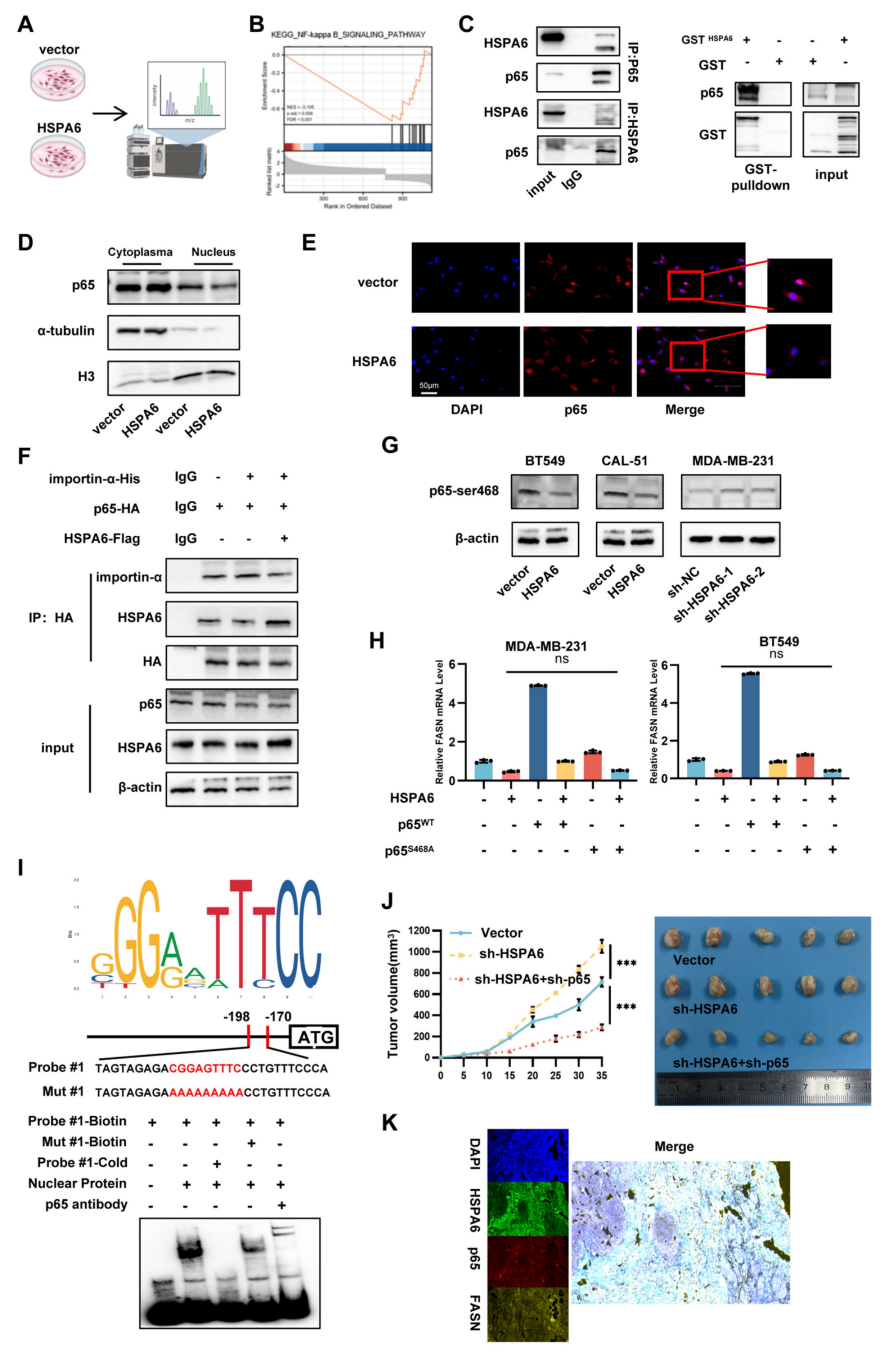
** HSPA6 inhibits translocation of p65 protein into nucleus in an importin-dependent manner.** The flowchart of LC-MS. **B)** GSEA showed a negative correlation between elevated HSPA6 expression and NF-κB pathway activation. **C)** Co-IP assays using an anti-p65/HSPA6 antibody confirmed the interaction between HSPA6 and p65, which was detected using an anti-p65/HSPA6 antibody (left). GST-pulldown assays using an anti-GST antibody to pull down HSPA6 confirmed its direct interaction with endogenous p65, as detected by an anti-p65 antibody (right). **D)** The nuclear and cytoplasmic extracts of HSPA6-overexpressing and control cells determined by Western blot. **E)** The location of p65 in HSPA6-overexpressing and control cells determined by immunofluorescence staining. **F)** The interaction between HSPA6, p65, and importin-α in TNBC cells determined by co-IP with an anti-HA(p65) antibody, followed by WB for indicated targets. **G)** Overexpression of HSPA6 descrepted the phosphorylation of p65 in ser468 determined by Western blot. **H)** The mRNA level of FASN was performed by using RT-qPCR in different groups. **I)** Prediction of the motif sequence of FASN via the JASPAR database. EMSA assay showed the binding ability of p65 with biotin-labeled oligonucleotides containing CGGAGTTTCC motif from FASN. **J)** Representative xenograft images from nude mice subcutaneously implanted with HSPA6-, p65/HSPA6-depleted MDA-MB-231 or control cells. Growth curve of the subcutaneous xenografts in each group was measured.** K)** Immunofluorescence staining for HSPA6 (green), p65 (red), FASN (orange) and DAPI (nucleus, blue) in TNBC tissues. Scale bar, 100 µm. (Data are presented as mean ± standard of error (SD) of three independent experiments. Statistical significance was determined using ANOVA with post-hoc Tukey multiple comparison, * P < 0.05, ** P < 0.01, *** P < 0.001, ns.: not significant.)

To elucidate the regulatory mechanism of HSPA6 on Lands cycle inhibition, Co-IP coupled with MS was employed to identify HSPA6-interacting proteins (Figure 6A). NF-κB, a widely reported transcription factor regulating cytokines and chemokines^45^, ^46^, emerged as a candidate pathway through GSEA of RNA sequencing data (Figure 6B). Specifically, p65 (RELA), a subunit requiring nuclear translocation for NF-κB activation, was directly co-precipitated with HSPA6 (Figure 6C). Given that p65 is a canonical transcription factor, it primarily functions by translocating to the nucleus^47^. Furthermore, the impact of HSPA6 on p65 subcellular localization was analyzed. Cell fractionation assays revealed that HSPA6 overexpression reduced the nuclear p65 accumulation (Figure 6D), a finding corroborated by immunofluorescence assays, which showed decreased nuclear p65 intensity upon HSPA6 overexpression(Figure 6E). Importin-α1 (KPNA2) and importin-β1 (KPNB1), critical mediators of classical nuclear import^48^, were identified as HSPA6-binding partners (Figure S6A). Importin α typically binds directly to cargo proteins bearing a nuclear localization signal (NLS) but lacks intrinsic nucleocytoplasmic shuttling activity; it therefore requires importin β for nuclear import. Meanwhile, Importin β possesses nucleocytoplasmic shuttling capability and, by forming a complex with importin α, mediates the transport of the entire complex through the nuclear pore complex into the nucleus. In the present study, HSPA6 inhibited the binding of p65 with importin-α (Figure 6F). Genetic perturbation experiments using p65-knockdown and NLS-truncated p65 constructs confirmed that HSPA6-mediated p65 nuclear exclusion was contingent upon functional import machinery (Figure S6B-C). Results showed that overexpression of importin-α promoted the nuclear localization of p65. Conversely, importin-α knockdown significantly attenuated the HSPA6 knockdown-induced enhancement of p65 nuclear localization, suggesting that importin-α is responsible for mediating the nuclear translocation of p65 (Figure 6D-E). Moreover, overexpression of p65 overexpression rescued the decreased colony formation capability resulting from HSPA6 overexpression, while NLS-truncated p65 failed to exert a similar effect (Figure S6F).

To investigate the mechanisms by which upstream factors regulate the nuclear function of p65, posttranslational modifications of p65 were examined. p65 phosphorylation plays a critical role in regulating NF-κB activity^49^. HSPA6 significantly decreased phosphorylation at Serine 468 of p65, whereas phosphorylation at Serine 564 of p65 remained unchanged (Figure 6G and S6G). To further investigate the mechanism of direct interaction between HSPA6 and p65, truncation mutants with different domains were constructed for HSPA6. Taking into account the literature and protein domain prediction websites, the NBD domain and SBD domain of HSPA6 were truncated and fused to express the Flag tag. HSPA6 full-length plasmid and two truncations of HSPA6 were transfected into 293T tool cells for Co-IP assays. By Co-IP assay, HSPA6 full-length and △NBD could immunoprecipitate the p65-s468 protein, however △SBD could not. It suggests that the SBD domain of HSPA6 directly interacts with the p65 protein (Figure S7F). Then, Serine 468 was mutated to alanine. Reconstitution with wild-type p65 rescued HSPA6-induced down-regulation of FASN expression, whereas phospho deficient p65^S468A^ failed to restore this phenomenon (Figure 6H and S6H). Thus, p65 phosphorylation and importin-mediated nuclear translocation are essential for HSPA6-induced down-regulation of FASN expression. TCGA data analysis revealed an inverse correlation between FASN and p65 expression in TNBC (Figure S6I). Public ChIP-seq datasets indicated p65 occupancy at the FASN promoter (Figure S6J), and we predicted that the CGGAGTTTCC motif (179 nt-188 nt) and the GGTGGTTTCC motif (1750 nt-1759 nt) in the FASN sequence are putative binding motifs for p65 (Figure 6I, Figure S6K). Furthermore, by performing EMSAs, we have verified that the CGGAGTTTCC motif of FASN is crucial for the interaction with p65. The motif specifically binds to p65, as indicated by the findings of the Supershift experiments. To identify specific binding elements in the FASN promoters, the binding sites were mutated to establish mutant promoter constructs. Subsequent luciferase assays revealed that mutations of the binding sites attenuated the positive effects of FASN and p65 on the luciferase activity of the mutant promoter construct (Figure S6L). ChIP-qPCR results confirmed that p65 indeed binds to the FASN promoter. Further analysis showed that, following HSPA6 overexpression, p65 binding to the FASN promoter was significantly reduced, and this inhibitory effect could be rescued by p65^WT^ but not by mutant p65^△NLS^ (Figure S6M). Moreover, overexpression of p65^WT^ partially rescued the transcriptional suppression of FASN caused by HSPA6 overexpression, with consistent changes observed in protein levels. In comparison, FASN expression in cells transfected with the p65^△NLS^ only partially restored FASN expression (Figure S6O). P65 Ser468 phosphorylation, which occurs mainly in the nucleus, primarily affects p65-dependent transactivation^50^. We observed that p65^WT^ significantly reversed the p65^△NLS^-induced decrease in FASN transcription, whereas the p65^S468A^ did not (Figure S6P). The results of immunofluorescence costaining of TNBC tissue further revealed co-localization of HSPA6, p65, and FASN (Figure 6K). These findings collectively signal that HSPA6 inhibits the occupancy of the promoter regions by p65 and promotes the transcriptional activity of p65. Functionally, p65 overexpression rescued HSPA6-mediated ferroptosis resistance (Figure S6N, S6Q). The effect of HSPA6 and p65 was investigated in the mouse xenograft tumor model. HSPA6 knockdown significantly increased tumor volume and weight, and accelerated tumor progression; however, the concurrent downregulation of P65 protein reversed these effects (Figure 6J). IHC staining revealed that tumors from sh-HSPA6 mice had up-regulated p65 and FASN, while concurrent downregulation of p65 reversed these effects (Figure S6R). These findings demonstrate that HSPA6 impairs nuclear translocation of p65 in an importin-dependent manner and reduces its phosphorylation at Ser468, thereby downregulating the mRNA expression of FASN.

### HSPA6-mediated metabolic reprogramming suppressed ANKIB1 palmitoylation

**Figure 7:**
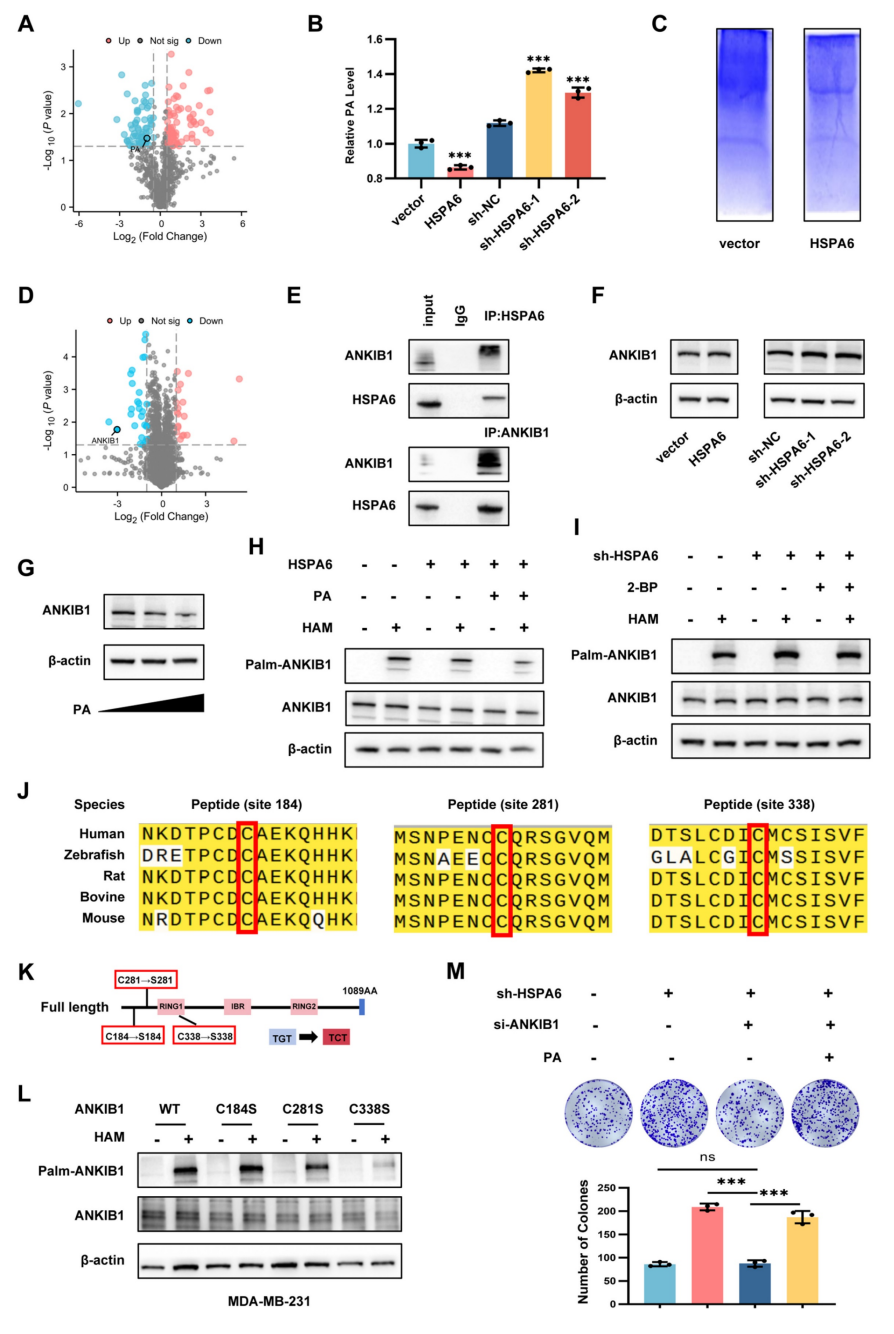
** HSPA6-mediated metabolic reprogramming suppresses ANKIB1 palmitoylation. A)** The volcano map shows different metabolites based on untargeted metabolomics. A red dot indicates a metabolomic with a high expression level, a blue dot indicates a metabolomic with a low expression level, while a grey dot represented metabolomic with no significance. **B)** The relative level of PA in HSPA6-overexpressing or HSPA6-depleted TNBC cells, as well as control cells was determined by ELISA. **C)** The blue-stained gel image illustrating the detection of protein palmitoylation levels using the ABE method. **D)** The volcano map shows different genes based on proteome in HSPA6-overexpressing cells compared to the control cells. Blue dots represent downregulated proteins, and red dots represent upregulated proteins. Proteins with |log2(fold change)| > 0.2 and -log10(p-value)>1.301 (dashed lines) were considered statistically significant. **E)** Co-IP assays using an anti-ANKIB1/HSPA6 antibody confirmed the interaction between HSPA6 and ANKIB1, which was detected using an anti-ANKIB1/HSPA6 antibody. **F)** The correlation between HSPA6 and ANKIB1 expression in TCGA databases. **G)** The expression of ANKIB1 in HSPA6-overexpressing or HSPA6-depleted TNBC cells, as well as the control cells determined by Western blot. **H)** The protein expression level of ANKIB1 in TNBC cells after PA treatment was determined by Western blot. **I)** The palmitoylation level of ANKIB1 in HSPA6-overexpressing TNBC cells with or without PA treatment was determined by Western blot. **J)** The palmitoylation of ANKIB1 in HSPA6-depleted TNBC cells with or without 2-BP treatment was determined by Western blot. **K)** Schematic representation of ANKIB1 and its potential palmitoylation site mutations. **L)** Western blot analysis of palmitoylation levels in cells transfected with ANKIB1 and its site-specific mutants (C184S, C281S, C338S). **M)** Colony formation analysis of HSPA6-depleted or/and ANKIB1-depleted TNBC cells with or without PA treatment. (Data are presented as mean ± standard of error (SD) of three independent experiments. Statistical significance was determined using ANOVA with post-hoc Tukey multiple comparison, * P < 0.05, ** P < 0.01, *** P < 0.001, ns.: not significant.)

Previous studies have identified palmitic acid (PA) as the primary substrate for protein palmitoylation^51^, ^52^. PA facilitates the metastatic progression of melanoma, breast cancer, and gastric cancer through a mechanism that is dependent on the CD36 fatty acid translocase receptor^53^. Given the reported roles of PA in promoting tumor growth and migration, we hypothesized that HSPA6 regulates TNBC via PA modulation. Metabolomic profiling showed that HSPA6 overexpression inhibited PA production (Figure 7A), which was further validated by Elisa quantification (Figure 7B). To identify HSPA6-dependent palmitoylation targets in TNBC progression, we performed acyl-biotin exchange (ABE) assay coupled with quantitative proteomic analysis (Figure 7C). A total of 145 differentially expressed proteins were identified, comprising 62 up-regulated and 83 down-regulated proteins (Figure 7D). Among these, ANKIB1, an E3 ubiquitin ligase, was selected for further investigation. Co-immunoprecipitation confirmed a direct interaction between HSPA6 and ANKIB1 (Figure 7E). Notably, HSPA6 did not alter total ANKIB1 mRNA expression (Figure S7A) or protein levels (Figure 7F). On the other hand, it modulated PA-dependent ANKIB1 degradation (Figure 7G). More importantly, HSPA6 overexpression attenuated ANKIB1 palmitoylation, whereas PA supplementation reversed this effect (Figure 7H), demonstrating that HSPA6 regulates ANKIB1 palmitoylation through metabolic reprogramming (Figure 7I). Then, ANKIB1-knockdown TNBC cells were constructed (Figure S7B). TCGA analysis indicated that ANKIB1 levels were higher in TNBC tumors compared to healthy tissues (Figure S7C). We tried to identify the cysteine residues of ANKIB1 that are S-palmitoylated by HSPA6. First, the motif-based prediction tool CSS-palm 4.0 predicted three palmitoylation sites in ANKIB1 (Cys184, 281, 338) in a sequence conserved across multiple species (Figure 7J). We then mutated the cysteine sites to serine (Figure 7K) and found that the mutation of 281 and 338 cysteines led to a significantly reduced palmitoylation signal of ANKIB1 compared with that of the wild-type in TNBC cells (Figure 7L, Figure S7D). Taken together, we prove that ANKIB1 is a palmitoylated substrate of HSPA6, with cysteines 281 and 338, serving as critical sites for palmitoylation. Moreover, ANKIB1 silencing exhibited significantly inhibited colony formation in HSPA6-knockdown TNBC cells, while PA partially restored the proliferative capacity attenuated by ANKIB1 silencing (Figure 7M). Taken together, these results demonstrate that HSPA6-mediated suppression of PA is essential for inhibiting ANKIB1 palmitoylation, thereby attenuating its E3 ubiquitin ligase activity.

### ANKIB1 induces the protein degradation and ubiquitination of HSPA6

**Figure 8:**
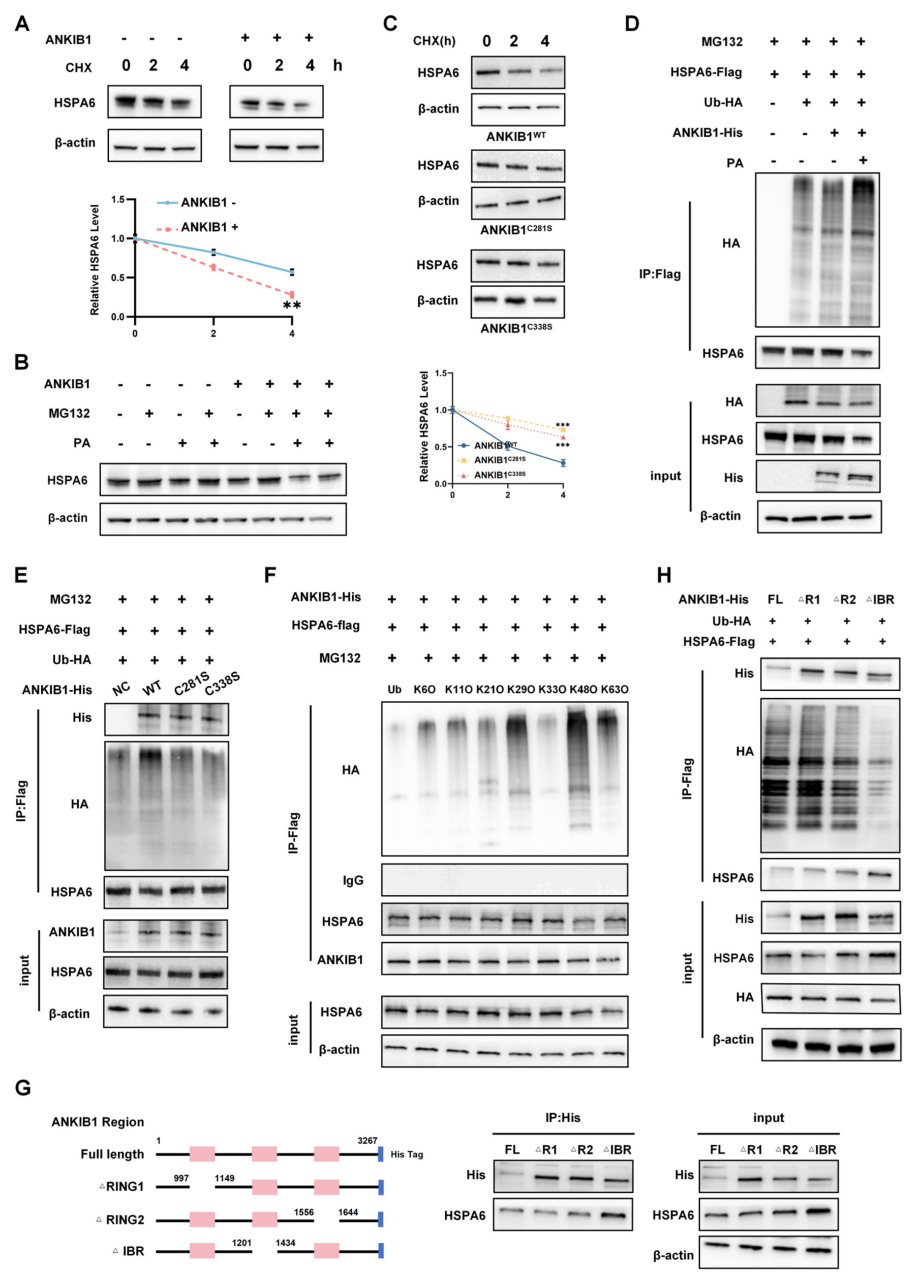
** ANKIB1 induces the protein degradation and ubiquitination of HSPA6.** The expression of HSPA6 in ANKIB1-overexpressing TNBC and control cells treated with CHX (50 µg/ml) for the indicated times determined by Western blot. **B)** ANKIB1-overexpressing and control cells were treated with MG132 (10 µm) or/and PA for 8 h. The cells were labeled and analyzed by Western blot. **C)** The protein expression level of HSPA6 in overexpressing ANKIB1^WT^, ANKIB1^C281S^, or ANKIB1^C338S^ cells treated with CHX (50 µg/ml) for the indicated times determined by Western blot.** D)** Co-IP assays using an anti-Flag antibody detect HSPA6 ubiquitination mediated by ANKIB1 or PA in 293T cells, which was detected by WB for indicated targets. **E)** Co-IP assays using an anti-Flag antibody detect HSPA6 ubiquitination mediated by ANKIB1^WT^, ANKIB1^C281S^, or ANKIB1^C338S^ in 293T cells, which was detected by WB for indicated targets. **F)** Co-IP assay using an anti-Flag antibody was applied to assess the ubiquitination level of HSPA6 affected by the seven types of ubiquitin mutated plasmids, which was detected by WB for indicated targets.** G)** Diagrammatic representation of ANKIB1 and its truncated forms. 293T cells were transfected with the indicated constructs subjected to immunoprecipitation with anti-His. **H)** Co-IP assays using an anti-Flag antibody detect HSPA6 ubiquitination mediated by ANKIB1 domains, which was detected by WB for indicated targets. Co-IP assay revealed IBR domains were required for the ubiquitination activity of ANKIB1. (Data are presented as mean ± standard of error (SD) of three independent experiments. Statistical significance was determined using ANOVA with post-hoc Tukey multiple comparison, * P < 0.05, ** P < 0.01, *** P < 0.001, ns.: not significant.)

As an E3 ubiquitin ligase, ANKIB1 catalyzes polyubiquitination to target substrates for proteasomal degradation^54^. To determine the regulatory effect of ANKIB1 on HSPA6 expression, cycloheximide (CHX) chase assays were performed. The results revealed that ANKIB1 overexpression accelerated HSPA6 degradation in TNBC cells (Figure 8A) while the half-life of HSPA6 protein was significantly longer in si-ANKIB1 cells compared to si-NC cells (Figure S7E), indicating that ANKIB1-mediated proteolysis. Consistent with this, proteasome inhibition by MG132 attenuated HSPA6 degradation and abrogated the effects of ANKIB1, while PA partially reversed ANKIB1-induced HSPA6 reduction (Figure 8B). Notably, ANKIB1 markedly reduced the stability of the HSPA6 protein, whereas this destabilizing effect was mitigated to some extent by the C281S or C338S mutation (Figure 8C). Given that ANKIB1 is an E3 ligase and that ANKIB1 may degrade HSPA6 via the proteasome pathway, ubiquitination assays were conducted to examine the effect of ANKIB1 on the ubiquitination of the HSPA6 protein, and the results showed that ANKIB1 increased the ubiquitination level of HSPA6, and that exogenous PA supplementation significantly enhanced the activity of ANKIB1, further promoting HSPA6 ubiquitination. (Figure 8D). Furthermore, mutation of both palmitoylation sites (C281S or C338S) significantly diminished the enhancing effect of ANKIB1 on HSPA6 ubiquitination, suggesting that the palmitoylation status of ANKIB1 affects its E3 ubiquitin ligase activity. These findings reveal a feedback loop in which the palmitoylation status of ANKIB1 governs its E3 ligase activity, thereby promoting HSPA6 degradation and subsequently influencing PA production, which in turn regulates ANKIB1 palmitoylation. To address whether ANKIB1 palmitoylation regulates ferroptosis sensitivity in TNBC by modulating HSPA6 protein stability, rescue experiments were performed by overexpressing ANKIB1^WT^ or its C281S and C338S mutants in HSPA6-knockdown TNBC cells. Consistent with our hypothesis, stable overexpression of ANKIB1^WT^ and the C281S or C338S mutants rescued the HSPA6 knockdown-induced decrease in ROS levels (Figure S8A) and MMP (Figure S8B). More importantly, the ferroptosis sensitivity in sh-HSPA6 cells were more profoundly reversed by ANKIB1^WT^ than in sh-HSPA6 cells transduced with C281S or C338S mutants. The ubiquitin molecule can form a polyubiquitin chain through linkage at one of its seven lysine residues (Lys6, Lys11, Lys27, Lys29, Lys33, Lys48, and Lys63). E3 ubiquitin ligase catalyzes the reaction, enabling the substrate to attach to the polyubiquitin chain, forming a polyubiquitinated substrate^55^, ^56^. To determine the specific type of ubiquitin chain involved, seven ubiquitin site mutant plasmids were constructed, including K48O (K48O ubiquitination refers to a ubiquitin construct in which all lysine residues, except for lysine 48, are mutated to arginine, allowing only the formation of K48-linked ubiquitin chains). The results demonstrated that ANKIB1 promoted HSPA6 ubiquitination via K48-linked ubiquitin chains (Figure 8F). Next, seven lysine (K) to arginine (R) mutants (K6R, K11R, K27R, K29R, K33R, K48R, K63R) of ubiquitin were constructed. The findings indicated that the K48R significantly diminished ANKIB1-induced HSPA6 ubiquitination (Figure S7H). These findings indicated that ANKIB1 facilitates the K48-linked ubiquitination of HSPA6.

We subsequently cotransfected full-length HSPA6-Flag plasmids with deletions in the SBD(ΔSBD) and NBD(ΔNBD) domains with the ANKIB1-His plasmid and performed ubiquitination and Western blot analyses (Figure S7F). By CO-IP assay, HSPA6 full-length and △NBD could immunoprecipitate the ANKIB1-His protein, however △SBD could not interact with ANKIB1, and ANKIB1 did not increase the ubiquitination level of HSPA6-ΔSBD group (Figure S7G). It suggests that the SBD domain of HSPA6 the domain directly interacts with the ANKIB1 protein. Taking into account the literature and protein domain prediction websites, the RING1 domain, RING2 domain and IBR domain of ANKIB1 were truncated and fused to express the His tag. ANKIB1 full-length plasmid and three truncations of ANKIB1 were co-transfected with HSPA6-Flag and Ub-HA into 293T tool cells for CO-IP assays (Figure 8G). It shows that the R1, R2, and IBR domains of ANKIB1 does not affect interaction with HSPA6 protein, while ANKIB1 did not increase the ubiquitination level of HSPA6 or decrease its total protein level in the ANKIB1-ΔIBR group (Figure 8H). These findings indicate that IBR domain of ANKIB1 binds to the HSPA6 protein and affects its ubiquitinated degradation.

### HSPA6 impedes the malignant progression of TNBC through reliance on HSPA6/p65/FASN/ANKIB1 axis

Given the potential of HSPA6/p65/FASN/ANKIB1 axis to regulate TNBC progression, we conducted functional analyses by using FASNi and HSPA6/ANKIB1 overexpression viruses to induce or inhibit ferroptosis, evaluating the role of this axis in TNBC cells. HSPA6 overexpression and FASNi synergistically reduce ANKIB1-induced ROS levels (Figure S8C) and elevated lipid peroxidation (Figure S8D), as well as changes in transwell migration (Figure S8E) and colony formation abilities (Figure S8F). Further investigations into the regulatory interaction in HSPA6/p65/FASN/ANKIB1 axis have underscored that HSPA6 and FASNi effectively mitigated ANKIB1-induced MDA depletion (Figure S8G) and iron overload (Figure S8I). Moreover, HSPA6 and FASNi also reversed ANKIB1-induced GSH depletion accumulation (Figure S8H) and mitochondrial morphological changes in TNBC cells (Figure S8J). Additionally, we utilized the TNBC xenograft-bearing mice model to investigate the *in vivo* effects of the HSPA6/p65/FASN/ANKIB1 regulatory axis. We delivered TVB-3166 targeting FASN, along with ANKIB1 or HSPA6 overexpression plasmids to TNBC mice model. FASNi causes a potent anti-tumor effect, which was attenuated by ANKIB1 overexpresison, and FASNi with HSPA6 overexpression combination treatment markedly suppressed tumor progression (Figure S8K-L).

## Discussion

TNBC remains a therapeutic challenge due to its aggressive phenotype and limited treatment option^57^. The process of cell death is intricately associated with both the initiation and development of tumors. Ferroptosis - a form of regulated cell death driven by iron-dependent lipid peroxidation - has emerged as a promising therapeutic vulnerability in TNBC, which frequently exhibits dysregulated iron metabolism, lipid accumulation, and ROS^58^. Induction of ferroptosis has been shown to inhibit the growth of TNBC cells. Nevertheless, the clinical translation of ferroptosis-targeted therapies remains constrained, emphasizing the imperative to develop more potent therapeutic strategies that exploit ferroptotic vulnerability for tumor suppression. Emerging evidence indicates that ferroptosis-related genes serve as robust indicators of patient prognosis and therapeutic response in cancer^29^, ^59^, ^60^. For instance, the expression statuses of ACSL4 and GPX4—respective positive and negative regulators of ferroptosis—serve as independent predictors of pathological complete response to neoadjuvant chemotherapy and prognostic factors for patient survival outcomes^61^. Identifying the novel driving genes of ferroptosis can provide valuable insights into TNBC pathogenesis and treatment.

This study initially examined the expression patterns of genes associated with ferroptosis in breast cancer tissues. We established a predictive model that stratified breast cancer cells into two distinct groups based on ferroptosis-related gene expression profiles, determining an optimal cutoff value for differentiation. From this analysis, HSPA6 emerged as a consistently differentially expressed gene across multiple ferroptosis subtypes, suggesting its potential role as a tumor suppressor in breast cancer pathogenesis. HSPs constitute a highly conserved family of molecular chaperones that are upregulated in response to various cellular stressors, including elevated temperatures, oxidative stress, and exposure to toxins, which can be attributed to their overexpression in numerous cancer types^62^. HSPA6 has been shown to play a decisive role in cancer progression, including tumorigenesis, metastasis, and multidrug resistance, by regulating key carcinogenesis-related proteins^23^, ^63^. Previous research has revealed that HSPA6 binds to SLC2A6, thereby inducing mitochondrial dysfunction which ultimately culminates in tumor cell death^64^. Corroborating this finding, our results demonstrate that low HSPA6 expression in TNBC tissues is associated with poor patient prognosis. Furthermore, we found that HSPA6 overexpression inhibits viability and promotes cytotoxicity in TNBC cells *in vitro*. Consistent with these *in vitro* findings, our animal studies confirmed that HSPA6 suppresses TNBC tumor growth *in vivo*. However, the exact mechanism underlying the function of HSPA6 remains to be elucidated.

Functionally, HSPA6 overexpression significantly induced ferroptosis while concomitantly inhibiting TNBC cell proliferation and migration in both *in vitro* and *in vivo* settings. Mounting evidence suggests that dysregulation of lipid metabolism contributes to cancer progression^65^, ^66^. Lipids play crucial roles in various biological processes within the body, such as providing energy storage, acting as signaling molecules, and serving as structural components of membranes^67^. According to a previous study, TNBC is hallmarked by elevated levels of lipids and iron, which predispose these tumors to ferroptosis^68^. Detailed metabolic and metabolomic investigations of the ferroptosis-sensitive subpopulation within TNBC have demonstrated a significant increase in intracellular PUFA levels, especially arachidonic acid and adrenic acid, suggesting their critical role in the ferroptotic process^69^. As ferroptosis is characterized by the accumulation of lethal lipid peroxides through the peroxidation of phospholipids containing PUFAs, recent studies have pointed out that reprogramming of lipid metabolism is closely related to ferroptosis sensitivity^70^-^72^. By performing non-targeted lipidomics assays, the results of this study demonstrated that HSPA6 remodels lipid metabolism by down-regulating FASN expression. The Lands cycle, also referred to as the phospholipid remodeling process, is the primary mechanism by which PLs are enzymatically remodeled to alter FA composition, thereby regulating membrane fluidity and function^17^. Cells utilize the Lands cycle to regulate the incorporation of PUFAs into PLs, thereby attenuating lipid peroxidation and decreasing the susceptibility to ferroptosis initiation^18^, ^73^. Notably, HSPA6 also disrupts the Lands cycle, a key phospholipid remodeling pathway that normally limits ferroptosis by controlling PUFA incorporation into membrane lipids. HSPA6 downregulates central Lands cycle enzymes, including LPCAT1 and cPLA2, leading to aberrant accumulation of PUFAs in phospholipids and enhancing their peroxidation susceptibility. Combined with FASN inhibition, which shifts cells toward exogenous PUFA uptake, HSPA6 engages a dual metabolic rewiring strategy that synergistically amplifies lipid peroxidation and ferroptosis. This coordinated suppression of both *de novo* lipogenesis and phospholipid remodeling represents a novel mechanism through which a molecular chaperon regulates membrane lipid composition to promote ferroptosis.

**Figure 9:**
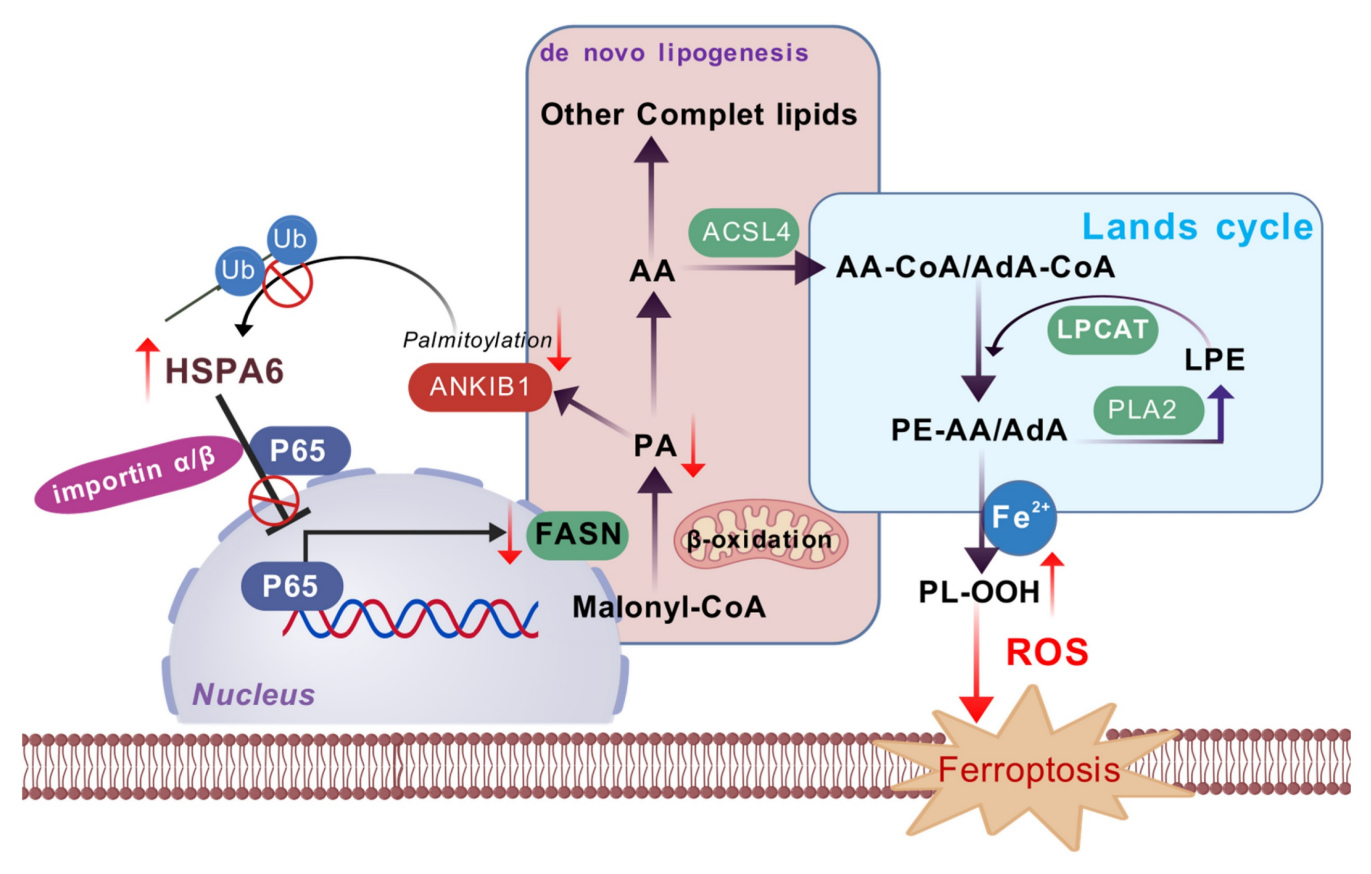
** A model of HSPA6-mediated ferroptosis in TNBC**.

The findings of this study collectively delineate a dynamic, self-amplifying regulatory circuit centered on HSPA6-mediated lipid metabolic reprogramming (Figure 9). The core mechanism of this program lies in the precise regulation of the transcription factor p65 by HSPA6. The NF-κB signaling pathway is critical for the regulation of ferroptosis responses in cancer and is recognized as a hallmark of cancer progression and a promising therapeutic target. To elucidate the intricate molecular mechanisms by which HSPA6 induces ferroptosis via regulating lipid metabolic reprogramming, we first investigated whether HSPA6 modulates the NF-κB signaling pathway and thereby exerts tumor-suppressive effects in breast cancer. HSPA6 directly binds to p65 via its SBD domain, which impedes the effective interaction between p65 and the importin α/β complex, thereby sequestering p65 in the cytoplasm. Phosphorylation of p65 at Ser468 occurs predominantly in the nucleus^74^. This interaction specifically inhibits phosphorylation at the Ser468 site of p65—a critical post-translational modification—whose suppression blocks p65 transcriptional activity. As p65 is a key transcriptional activator of the FASN gene, its inhibited function directly leads to the downregulation of FASN, the engine driving *de novo* lipogenesis. This step not only cuts off the endogenous supply of saturated fatty acids such as PA, but also cooperatively downregulates key enzymes of the Lands cycle (LPCAT1 and cPLA2). Together, these effects result in an abnormal increase in the proportion of polyunsaturated fatty acids (PUFAs) in membrane phospholipids, thereby providing ample substrate for the lipid peroxidation required for ferroptosis.

Furthermore, this study reveals a sophisticated post-translational feedback loop. HSPA6-mediated suppression of FASN leads to decreased levels of its key product, PA. Accumulating scientific evidence indicates that small-molecule metabolites play a central role in modulating the epigenetic regulation of proteins, thereby influencing various cellular processes through post-translational modifications and chromatin remodeling mechanisms^75^. PA has been demonstrated to modulate protein palmitoylation, a post-translational modification that plays an integral role in regulating various aspects of protein function^76^. As the primary substrate for protein palmitoylation, reduced PA directly attenuates the palmitoylation modification of the E3 ubiquitin ligase ANKIB1. Functionally impaired ANKIB1 exhibits a significantly diminished capacity to catalyze K48-linked polyubiquitination and subsequent degradation of HSPA6, thereby establishing a positive feedback cycle. This cycle ensures that under sustained stress conditions, HSPA6 protein stability is maintained or even enhanced, allowing its inhibitory effect on the p65/FASN pathway and its reprogramming of lipid metabolism to persist and amplify.

This model not only mechanistically explains why TNBC patients with high HSPA6 expression have a more favorable prognosis but also provides a clear roadmap for designing combination therapies that target this metabolic vulnerability. For instance, FASN inhibitors (e.g., TVB-3166) may synergize with this axis to further block residual lipid synthesis, thereby more effectively inducing ferroptosis and overcoming therapy resistance.

## Conclusion

In summary, this work systematically elucidates that HSPA6, as a crucial stress-responsive factor, converts cellular stress signals into sustained lipid metabolic reprogramming and ferroptosis activation in TNBC by constructing the HSPA6/p65/FASN/ANKIB1 regulatory axis. This role transcends its conventional function as a molecular chaperone, unveiling its novel function as a metabolic checkpoint in tumors, and provides a new theoretical foundation and potential combination therapeutic targets for leveraging ferroptosis against TNBC.

## Supplementary Material

Supplementary figures and tables.

## Data Availability

All data supporting the findings of this study are available from the corresponding authors upon reasonable request.

## Abbreviations

TNBC: triple-negative breast cancer
HSPA6: Heat shock 70-kDa protein 6
FASN: Fatty Acid Synthase
PUFAs: polyunsaturated fatty acids
ER: estrogen receptor
PR: progesterone receptor
HER2: human epidermal growth factor receptor 2
GPX4: glutathione peroxidase 4
GSH: glutathione
ROS: reactive oxygen species
FAs: Fatty acids
SFAs: saturated
MUFAs: monounsaturated
