# Supplementary material for: HSPA6 Promotes Ferroptosis in Triple-Negative Breast Cancer by Rewiring Lipid Metabolism to Potentiate Membrane Lipid Peroxidation
Source: Int J Biol Sci. 2026 Apr 8;22(8):4168–93. doi: 10.7150/ijbs.129745 (PMC13137960; doi:10.7150/ijbs.129745)
Supplement: Supplementary file 1 — Supplementary figures and tables. [file ijbsv22p4168s1.pdf]

Figure S1：HSPA6 is a crucial suppressor protein associated with ferroptosis in TNBC.

A) The volcano map shows that ferroptosis-related genes are expressed differently in BRCA and non-cancer samples. A red dot indicates a gene with a high expression level, a blue dot indicates a gene with a low expression level, while a grey dot represented genes with no significance. **B)** The heat maps were generated to visualize key ferroptosis-related DEGs. **C)** A univariate Cox regression analysis was performed on 11 prognostic ferroptosis-related genes associated with overall survival . **D)** The optimal parameter (λ) was determined by using the minimum criteria set by the vertical lines in the LASSO Cox regression model. **E)** The LASSO coefficient of the LRGs signature. **F)** The gene set was optimized using regression coefficient calculations. **G)** Overall survival of BRCA patients based on ferroptosis-related score group by Kaplan–Meier analysis from the TCGA. **H)** Pie charts showing the Chi-squared test of clinicopathologic factors for ferroptosis-related score group in BRCA tumor samples from the TCGA. **I)** A comprehensive nomogram for predicting the survival probabilities of BRCA patients at 1-year, 3-year, and 5-year intervals based on ferroptosis score and clinicopathologic. **J)** Overall survival of BRCA patients based on ferroptosis-related score group by Kaplan–Meier analysis from the GSE58812 and GSE65216. **K)** Venn diagram exhibiting DEGs in ferroptosis score group in the TCGA, GSE58812, and GSE65216. **L)** Analysis of HSPA6 expression in samples with different grade and different prognosis from GSE31519. **M)** HSPA6 staining of unpaired BRCAs from Human Protein Atlas (HPA) database. (Data are presented as mean ± standard of error (SD) of three independent experiments. Statistical significance was determined using ANOVA with post-hoc Tukey multiple comparison, * $P < 0.05$, ** $P < 0.01$, *** $P < 0.001$, ns.: not significant.)

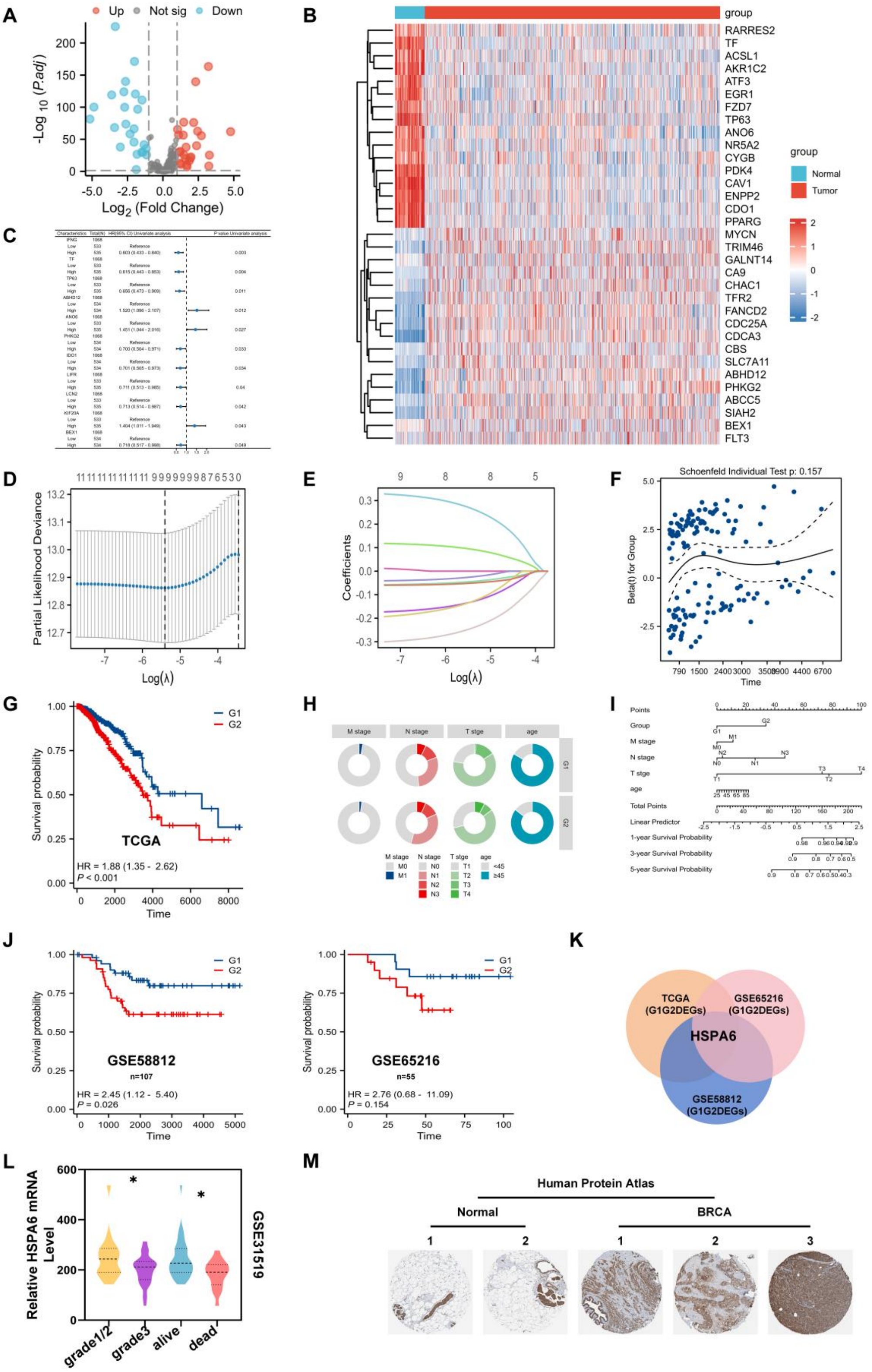
A
Up
Not sig
Down
-Log 10 (P.adj)
Log2 (Fold Change)
B
group
RARRES2
TF
ACSL1
AKR1C2
ATF3
EGR1
FZD7
TP63
ANO6
NR5A2
CYGB
PDK4
CAV1
ENPP2
CDO1
PPARG
MYCN
TRIM46
GALNT14
CA9
CHAC1
TFR2
FANCD2
CDC25A
CDCA3
CBS
SLC7A11
ABHD12
PHKG2
ABCC5
SIAH2
BEX1
FLT3
Normal
Tumor
C
D
Partial Likelihood Deviance
Log(λ)
E
Coefficients
Log(λ)
F
Schoenfeld Individual Test p: 0.157
Beta(t) for Group
Time
G
G1
G2
Survival probability
TCGA
HR = 1.88 (1.35 - 2.62)
P < 0.001
Time
H
M stage
N stage
T stge
age
G1
G2
M0
M1
N0
N1
N2
N3
T1
T2
T3
T4
<45
≥45
I
Points
Group
M stage
N stage
T stge
age
Total Points
Linear Predictor
1-year Survival Probability
3-year Survival Probability
5-year Survival Probability
J
Survival probability
GSE58812
n=107
HR = 2.45 (1.12 - 5.40)
P = 0.026
Time
GSE65216
n=55
HR = 2.76 (0.68 - 11.09)
P = 0.154
Time
K
TCGA (G1G2DEGs)
GSE65216 (G1G2DEGs)
HSPA6
GSE58812 (G1G2DEGs)
L
Relative HSPA6 mRNA Level
GSE31519
grade1/2
grade3
alive
dead
M
Human Protein Atlas
Normal
BRCA
1
2
1
2
3

Figure S2：HSPA6 suppressed growth and metastasis of TNBC in vitro and in vivo.

A) Western blot was used to validate the knockdown of HSPA6 in TNBC cells. **B)** Representative images of plate colony formation assays after interferencing of HSPA6 in MDA-MB-231, BT549 cells and the corresponding statistical graphs (n = 3). **C)** Representative images of transwell migration assays after the downexpression of HSPA6 in TNBC cells and the corresponding statistical graphs (n = 3). **D)** The expression levels of EMT-related biomarkers, including E-cadherin, N-cadherin, and Vimentin in MDA-MB-231 cells detected by Western blot. **E)** The viability of HSPA6-downexpressing and control cells determined by CCK-8 assays. **F)** Wound healing assay demonstrated that HSPA6 inhibits TNBC cell migration, (n=3). **G)** Bioluminescent imaging of metastasis for xenograft mice after tail vein injection of cells. **H)** The viability of TNBC cells with HSPA6 knockdown was determined by EdU assay. (Data are presented as mean ± standard of error (SD) of three independent experiments. Statistical significance was determined using ANOVA with post-hoc Tukey multiple comparison, * $P < 0.05$, ** $P < 0.01$, *** $P < 0.001$, ns.: not significant.)

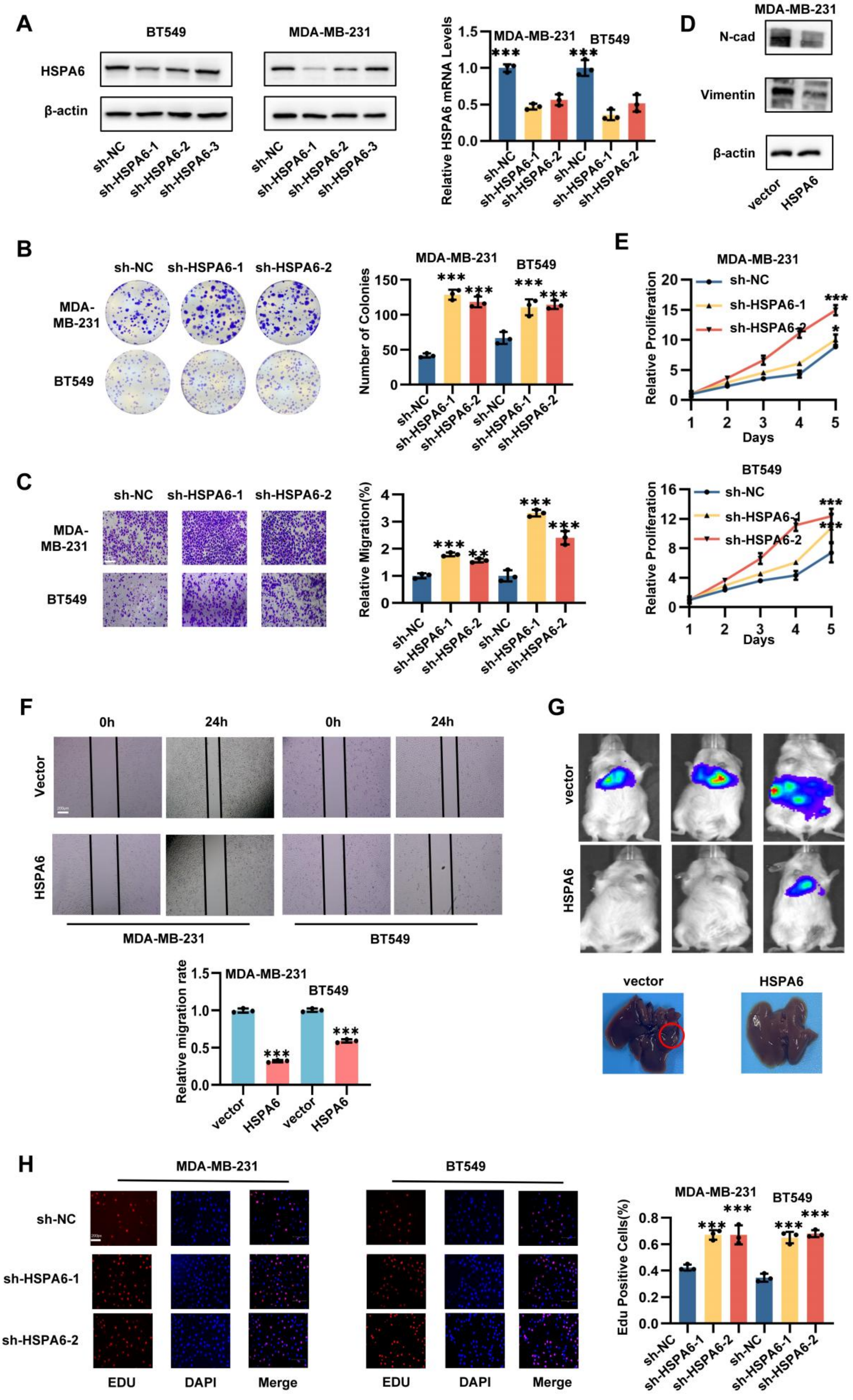
A
BT549
MDA-MB-231
HSPA6
β-actin
sh-NC
sh-HSPA6-1
sh-HSPA6-2
sh-HSPA6-3
Relative HSPA6 mRNA Levels
MDA-MB-231
BT549
***
D
MDA-MB-231
N-cad
Vimentin
β-actin
vector
HSPA6
B
sh-NC
sh-HSPA6-1
sh-HSPA6-2
MDA-MB-231
BT549
Number of Colonies
E
MDA-MB-231
Relative Proliferation
Days
BT549
C
Relative Migration(%)
F
0h
24h
Vector
HSPA6
MDA-MB-231
BT549
Relative migration rate
G
vector
HSPA6
H
MDA-MB-231
BT549
EDU
DAPI
Merge
Edu Positive Cells(%)

Figure S3：HSPA6 induces ferroptosis of TNBC in vitro and in vivo.

A) The viability of TNBC cells with different drug level was determined by CCK-8 assays (n = 5). **B-C)** The mRNA (B) and protein (C) expression levels of ferroptosis-related biomarker. **D)** Intracellular GSSG/GSH level of TNBC cells were measured by corresponding assay kits. **E)** Colony formation analysis of HSPA6-overexpressing TNBC cells and control cells treated with Fer-1 or Erastin. **F)** The expression of HSPA6, Ki67, ACSL4, FTH1, E-cadherin, and N-cadherin in xenografts of each group were assessed by immunochemistry. Scale bar, 100 μm. **G)** The expression of ACSL4 and FTH1 in TNBC tissues with altered HSPA6 expression levels. Scale bars, 100 μm. **H)** Immunofluorescence staining for HSPA6 (green), ACSL4 (red), FTH1 (orange) and DAPI (nucleus, blue) in TNBC tissues. Scale bar, 100 μm. (Data are presented as mean ± standard of error (SD) of three independent experiments. Statistical significance was determined using ANOVA with post-hoc Tukey multiple comparison, * $P < 0.05$, ** $P < 0.01$, *** $P < 0.001$, ns.: not significant.)

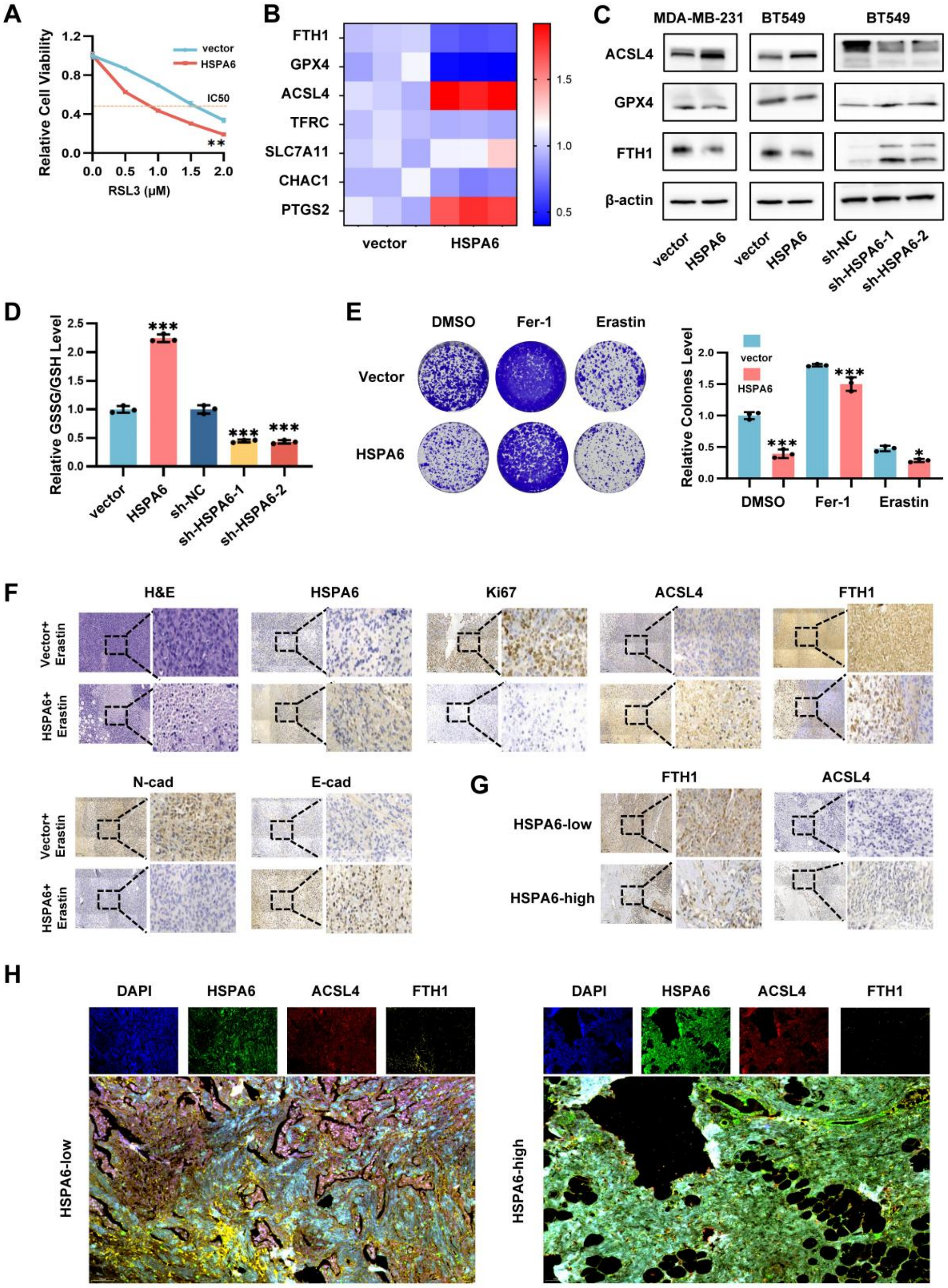
A
Relative Cell Viability
vector
HSPA6
IC50
RSL3 (μM)
B
FTH1
GPX4
ACSL4
TFRC
SLC7A11
CHAC1
PTGS2
vector
HSPA6
C
MDA-MB-231
BT549
BT549
ACSL4
GPX4
FTH1
β-actin
vector
HSPA6
vector
HSPA6
sh-NC
sh-HSPA6-1
sh-HSPA6-2
D
Relative GSSG/GSH Level
vector
HSPA6
sh-NC
sh-HSPA6-1
sh-HSPA6-2
E
DMSO
Fer-1
Erastin
Vector
HSPA6
Relative Colones Level
vector
HSPA6
DMSO
Fer-1
Erastin
F
H&E
HSPA6
Ki67
ACSL4
FTH1
Vector+ Erastin
HSPA6+ Erastin
N-cad
E-cad
Vector+ Erastin
HSPA6+ Erastin
G
FTH1
ACSL4
HSPA6-low
HSPA6-high
H
DAPI
HSPA6
ACSL4
FTH1
HSPA6-low
DAPI
HSPA6
ACSL4
FTH1
HSPA6-high

Figure S4：HSPA6 inhibits de novo lipogenesis and Lands cycles.

A) TNBC cells were treated with FASNi (0, 1, 2 μm) for 48 h for Western blot analyses. **B)** Viability assay of human TNBC cells with FASNi treated detected by CCK8. **C)** The FASN expression were detected following sh-HSPA6 and FASN i transfection. **D)** Oil red O staining for lipid droplets in TNBC cells. **E-F)** Intracellular TG levels (E) and CHOL (F) of TNBC cells were measured by corresponding assay kits. **G)** Intracellular lipid peroxidation and ROS content of TNBC cells were quantified by flow cytometry. **H)** Mitochondrial membrane potential was measured using the JC-1 probe. The distribution of JC-1 aggregates (PE channel) and monomers (FITC channel) was determined by flow cytometry. **I)** Representative images of plate colony formation assays after the knockdown of HSPA6 or treated with FASNi in TNBC cells and the corresponding statistical graphs (n = 3). **J, K, L)** Intracellular GSH levels (J), NAD^+/NADPH levels (K) and MDA levels (L) of TNBC cells were measured by corresponding assay kits. **M)** Incorporation of arachidonic acid (AA) alkyne in the indicated cell lines treated as indicated. (Data are presented as mean ± standard of error (SD) of three independent experiments. Statistical significance was determined using ANOVA with post-hoc Tukey multiple comparison, * $P < 0.05$, ** $P < 0.01$, *** $P < 0.001$, ns.: not significant.)

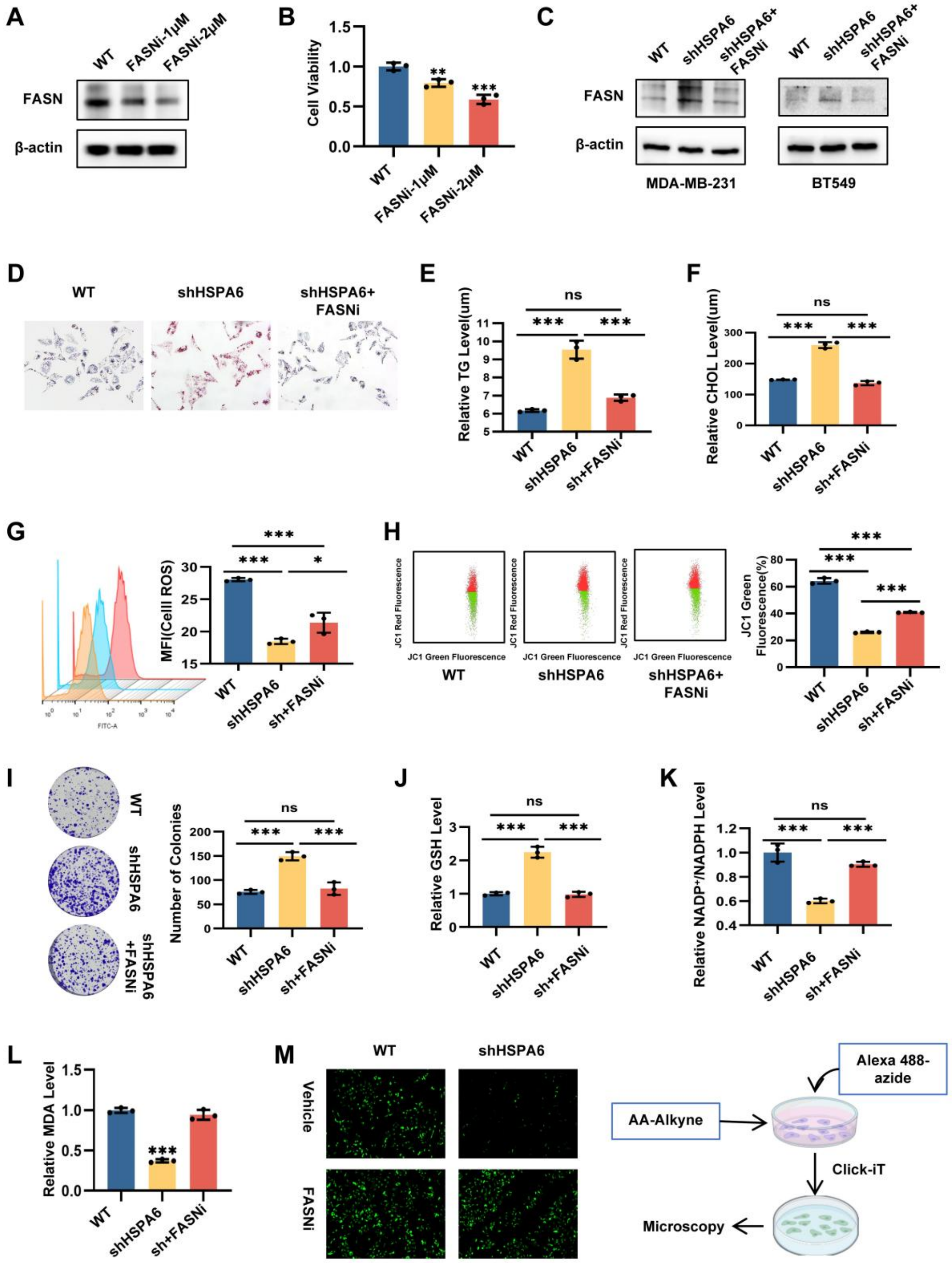
A
WT
FASNi-1μM
FASNi-2μM
FASN
β-actin
B
Cell Viability
C
shHSPA6
shHSPA6+ FASNi
MDA-MB-231
BT549
D
E
Relative TG Level(um)
sh+FASNi
F
Relative CHOL Level(um)
G
MFI(Celll ROS)
FITC-A
H
JC1 Red Fluorescence
JC1 Green Fluorescence
JC1 Green Fluorescence(%)
I
Number of Colonies
J
Relative GSH Level
K
Relative NADP+/NADPH Level
L
Relative MDA Level
M
Vehicle
FASNi
Alexa 488-azide
AA-Alkyne
Click-iT
Microscopy

Figure S5：HSPA6 inhibits de novo lipogenesis and Lands cycles.

A) Representative LPCAT1, cPLA2, and p-cPLA2 IHC staining of different HSPA6 level tissues from TMUCIH. Scale bars, 100 μm. **B)** Representative images of LPCAT1, cPLA2, and p-cPLA2 IHC staining xenografts. Scale bar, 100μm. **C)** Representative images of immunofluorescence staining for HSPA6 (green), LPCAT1 (red), and DAPI (nucleus, blue) in tissue sections from TNBC patients. Scale bar: 100 μm. (Data are presented as mean ± standard of error (SD) of three independent experiments. Statistical significance was determined using ANOVA with post-hoc Tukey multiple comparison, * P < 0.05, ** P < 0.01, *** P < 0.001, ns.: not significant.)

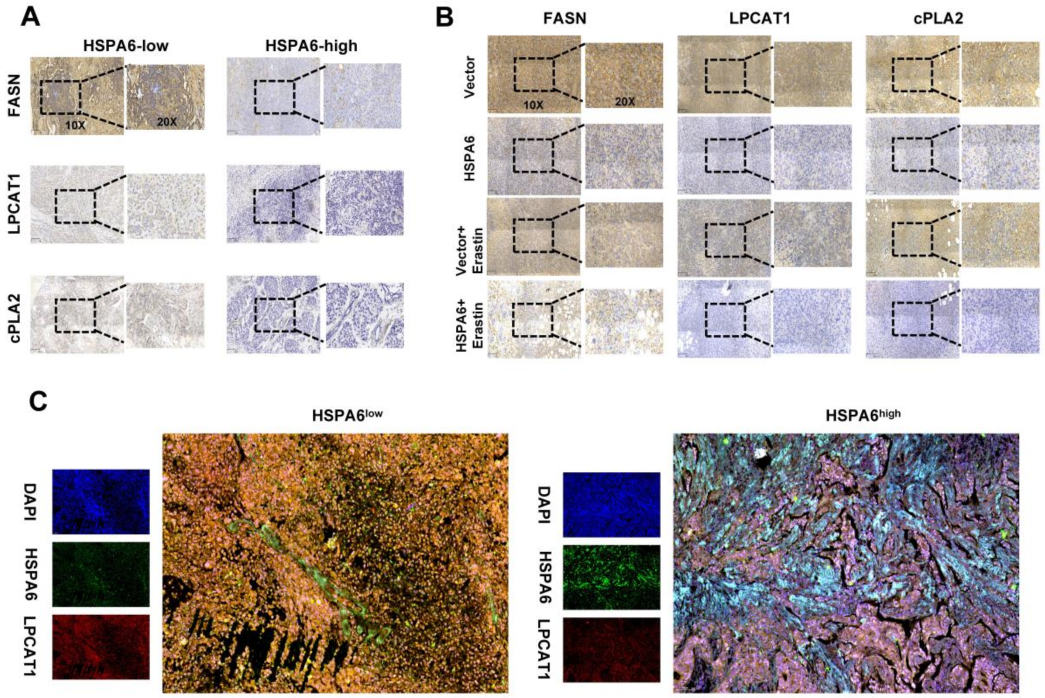

Figure S6：HSPA6 inhibits translocation of p65 protein into nucleus in an importin-dependent manner.

A) Co-IP assays using an anti-HSPA6 antibody detect the interaction between endogenous HSPA6 and importin-α，importin-β with HSPA6 in TNBC cells. **B)** The wild-type p65 ($p65^{WT}$) and a NLS-mutated p65 ($p65^{\Delta NLS}$) were transfected into TNBC cells and the localization of p65 was determined by Western blot. **C)** The expression level of p65 in TNBC cells was determined by Western blot. **D)** The vectors with wild-type p65 ($p65^{WT}$) and a mutation ($p65^{\Delta NLS}$) that disrupted p65 translocation into the nucleus were transfected into TNBC cells. **E)** The subcellular localization of p65 was determined by immunofluorescence staining. **F)** Colony formation analysis of $p65^{WT}$ or $p65^{\Delta NLS}$-overexpressing TNBC cells, as well as the control cells. **G)** The overexpression of HSPA6 does not altered the phosphorylation status of p65-Ser564. **H)** The mRNA level of FASN was performed by using RT-qPCR in different groups. I**)** The relationship between p65 and FASN from TCGA BRCA data. **J)** Public ChIP-seq datasets indicated p65 occupancy at the FASN promoter. **K)** EMSA assay showed the binding ability of p65 with biotin-labeled oligonucleotides containing GGTGGTTTCC motif from FASN. **L)** In 293T cells,P65 increased the promoter luciferase activity of FASN. The luciferase activity of the reporter gene was normalized to that of Renilla luciferase. **M)** ChIP-qPCR were used to verify the binding of p65 to the FASN promoter (up). ChIP-qPCR analysis of p65 binding at the FASN promoter upon HSPA6/$p65^{WT}$/$p65^{\Delta NLS}$ overexpression (down). **N)** The expression levels of ferroptosis-related biomarkers, including GPX4, FTH1, and ACSL4 in TNBC cells detected by western blot. **O)** RT-qPCR and western blot were used to detect the rescue effect of HSPA6 upregulation on FASN knockdown induced by T $p65^{WT}$/$p65^{\Delta NLS}$ overexpression. **P)** RT-qPCR and western blot were used to detect the rescue effect of HSPA6 upregulation on FASN knockdown induced by T $p65^{WT}$/$p65^{\Delta NLS}$/$p65^{S468A}$ overexpression. **Q)** The expression levels of ferroptosis-related biomarkers, including GPX4, FTH1, and ACSL4 in TNBC cells detected by western blot. **R)** The expression of H&E, p65, and FASN of each group were assessed by immunochemistry. Scale bar, 100 μm. (Data are presented as mean ± standard of error (SD) of three independent experiments. Statistical significance was determined using ANOVA with post-hoc Tukey multiple comparison, * $P < 0.05$, ** $P < 0.01$, *** $P < 0.001$, ns.: not significant.)

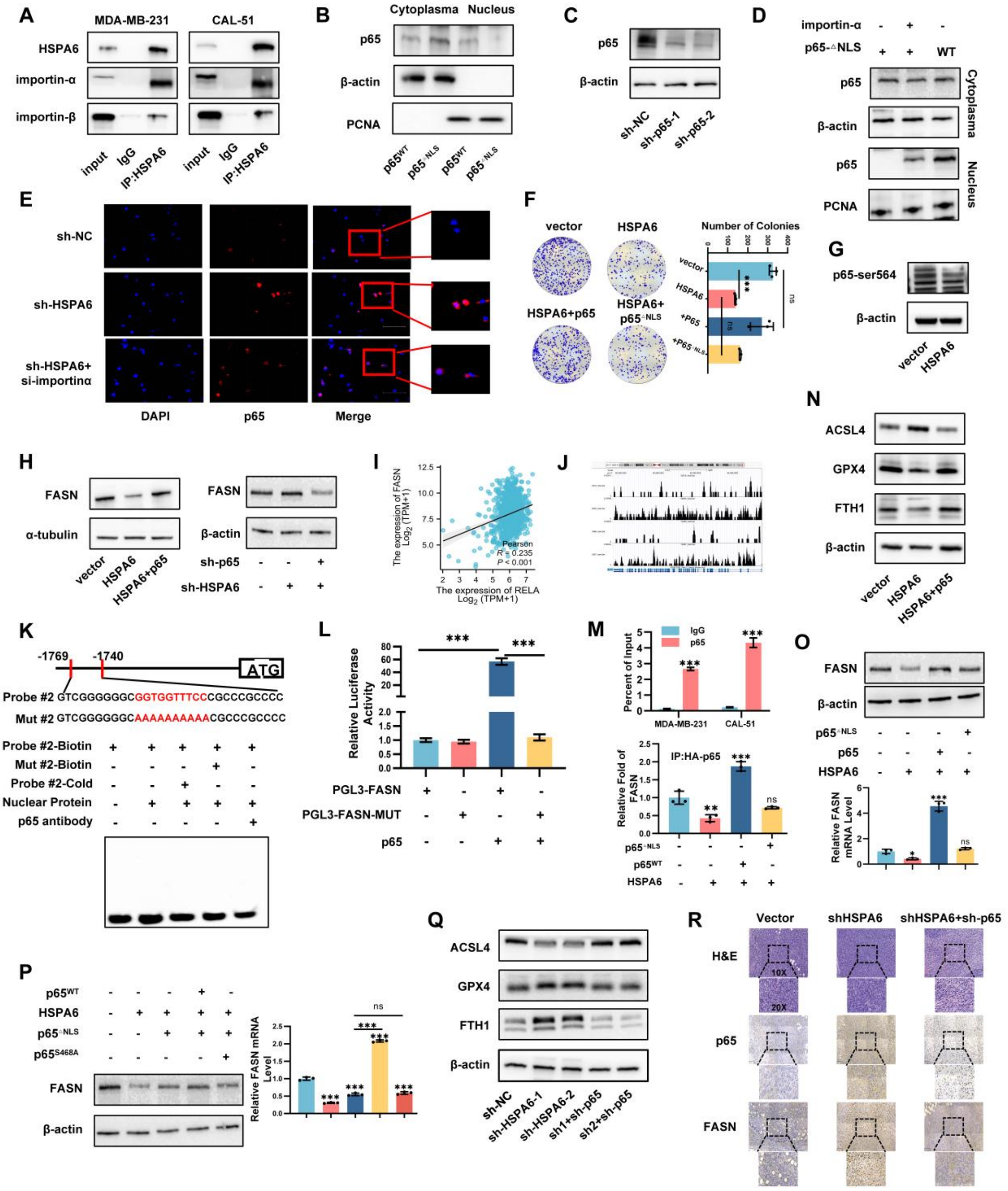
A
MDA-MB-231
CAL-51
HSPA6
importin-α
importin-β
input
IgG
IP:HSPA6
B
Cytoplasma
Nucleus
p65
β-actin
PCNA
C
sh-NC
sh-p65-1
sh-p65-2
D
importin-α
WT
Cytoplasma
Nucleus
E
sh-NC
sh-HSPA6
sh-HSPA6+
si-importinα
DAPI
Merge
F
vector
HSPA6
HSPA6+p65
HSPA6+
Number of Colonies
ns
G
p65-ser564
H
FASN
α-tubulin
HSPA6+p65
sh-p65
sh-HSPA6
I
The expression of FASN
The expression of RELA
Pearson
R = 0.235
P < 0.001
J
N
ACSL4
GPX4
FTH1
K
-1769
-1740
ATG
Probe #2 GTCGGGGGGCGGTGGTTTCCCGCCCGCCCC
Mut #2 GTCGGGGGGCAAAAAAAAAACGCCCGCCCC
Probe #2-Biotin
Mut #2-Biotin
Probe #2-Cold
Nuclear Protein
p65 antibody
L
Relative Luciferase Activity
PGL3-FASN
PGL3-FASN-MUT
M
IgG
Percent of Input
IP:HA-p65
Relative Fold of FASN
O
Relative FASN mRNA Level
P
Relative FASN mRNA Level
Q
sh-HSPA6-1
sh-HSPA6-2
sh1+sh-p65
sh2+sh-p65
R
Vector
shHSPA6
shHSPA6+sh-p65
H&E
10X
20X

Figure S7：HSPA6 functions through the SBD domain combined with the p65.

A) Correlation between HSPA6 and ANKIB1 expression in public BRCA databases. **B)** Western blot was used to validate the knockdown of ANKIB1 in TNBC cells. **C)** The expression of ANKIB1 in breast cancer and adjacent-healthy tissues from TCGA database. **D)** Western blot analysis of palmitoylation levels in cells transfected with ANKIB1 and its site-specific mutants (C184S, C281S, C338S). **E)** The expression of HSPA6 in si-ANKIB1 TNBC and control cells treated with CHX (50 μg/ml) for the indicated times determined by Western blot. **F)** Diagrammatic representation of HSPA6 and its truncated forms. 293T cells were transfected with the indicated constructs subjected to immunoprecipitation with anti-Flag, which was detected by WB for indicated targets. **G)** Co-IP assays using an anti-Flag antibody detect HSPA6 and its domains ubiquitination mediated by ANKIB1, which was detected by WB for indicated targets.Co-IP assay revealed SBD domains were required for the ubiquitination activity of HSPA6. **H)** Co-IP assay using an anti-Flag antibody was applied to assess the ubiquitination level of HSPA6 affected by the Ub-HA or Ub-K48R plasmids, which was detected by WB for indicated targets. (Data are presented as mean ± standard of error (SD) of three independent experiments. Statistical significance was determined using ANOVA with post-hoc Tukey multiple comparison, * $P < 0.05$, ** $P < 0.01$, *** $P < 0.001$, ns.: not significant.)

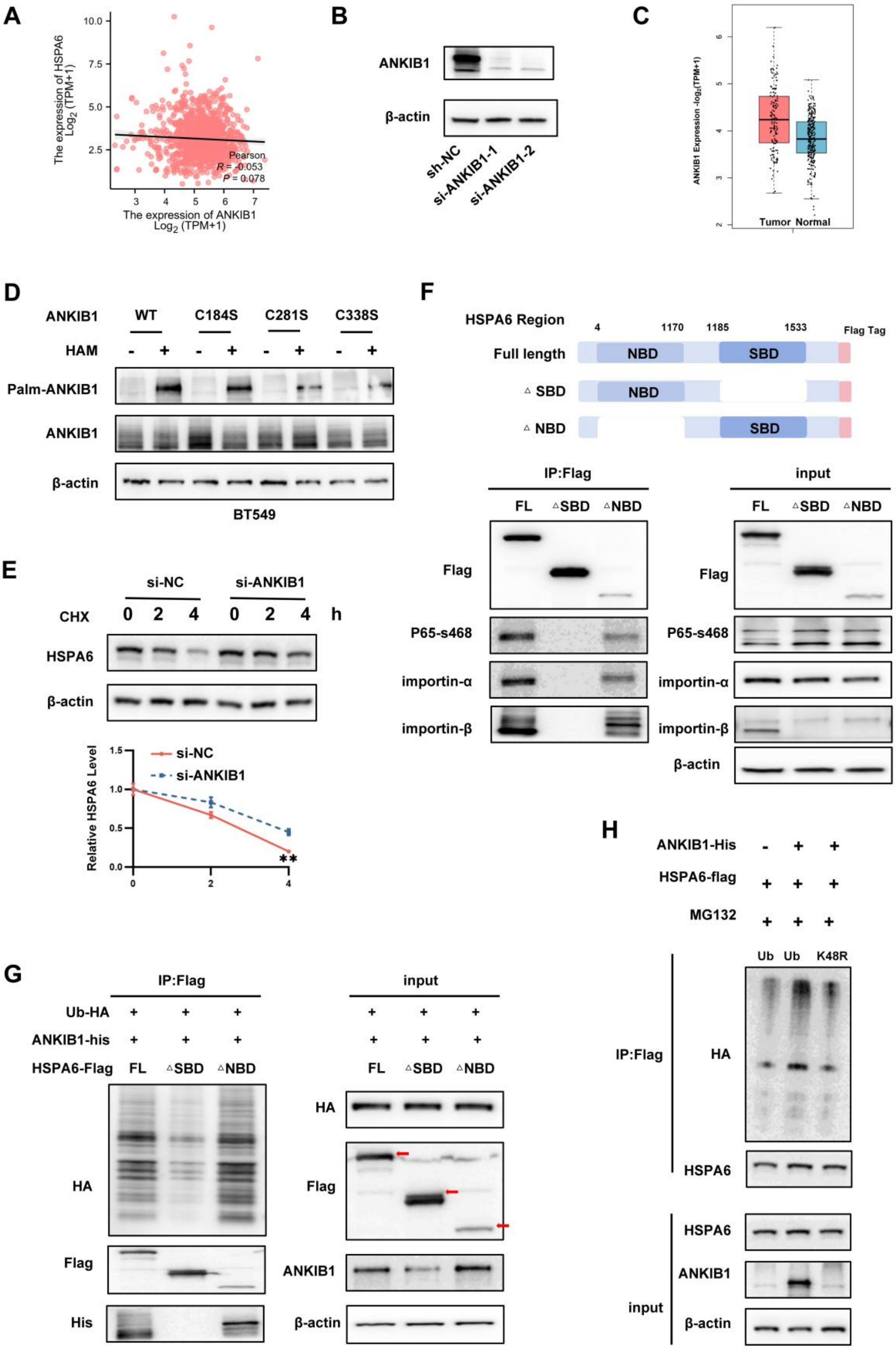
A
The expression of HSPA6
Log2 (TPM+1)
The expression of ANKIB1
Log2 (TPM+1)
Pearson
R = -0.053
P = 0.078
B
ANKIB1
β-actin
sh-NC
si-ANKIB1-1
si-ANKIB1-2
C
ANKIB1 Expression -log2(TPM+1)
Tumor
Normal
D
ANKIB1
WT
C184S
C281S
C338S
HAM
Palm-ANKIB1
ANKIB1
β-actin
BT549
E
si-NC
si-ANKIB1
CHX
h
HSPA6
β-actin
Relative HSPA6 Level
si-NC
si-ANKIB1
**
F
HSPA6 Region
Full length
△ SBD
△ NBD
NBD
SBD
Flag Tag
IP:Flag
input
FL
△SBD
△NBD
Flag
P65-s468
importin-α
importin-β
β-actin
G
IP:Flag
input
Ub-HA
ANKIB1-his
HSPA6-Flag
HA
Flag
His
ANKIB1
β-actin
H
ANKIB1-His
HSPA6-flag
MG132
Ub
K48R
IP:Flag
HA
HSPA6
ANKIB1
β-actin
input

Figure S8：HSPA6 facilitates ferroptosis by inhibiting the p65/FASN/ANKIB1 pathway in vitro

A) Intracellular ROS levels in overexpressing ANKIB1^{WT}, ANKIB1^{C281S}, or ANKIB1^{C338S} TNBC cells were detected by flow cytometry using DCFH-DA probe. **B)** Mitochondrial membrane potential was measured using the JC-1 probe. The distribution of JC-1 aggregates (PE channel) and monomers (FITC channel) in each group TNBC cells were determined by flow cytometry. **C)** Intracellular ROS levels in TNBC cells were detected by flow cytometry using DCFH-DA probe. **D)** Mitochondrial membrane potential was measured using the JC-1 probe. The distribution of JC-1 aggregates (PE channel) and monomers (FITC channel) in each group TNBC cells were determined by flow cytometry. **E)** Transwell assays to assess the effect of FASNi, ANKIB1, and HSPA6 on TNBC cell invasion. **F)** Clonogenic assays to examine the effects of each group on TNBC cell proliferation. **G)** The Intracellular MDA level were measured by corresponding assay kits. **H)** The Intracellular GSH level were measured by corresponding assay kits. **I)** The Intracellular Iron content were measured by corresponding assay kits. **J)** Transmission electron microscopy was used to detect the morphological changes in cellular mitochondria, including smaller size, reduced cristae, and even membrane rupture. Scale bars, 100 μm. **K)** Representative xenograft images from nude mice subcutaneously implanted with different group cells. **L)** Representative images of H&E staining staining of xenografts. Scale bar, 100 μm. (Data are presented as mean ± standard of error (SD) of three independent experiments. Statistical significance was determined using ANOVA with post-hoc Tukey multiple comparison, * $P < 0.05$, ** $P < 0.01$, *** $P < 0.001$, ns.: not significant.)

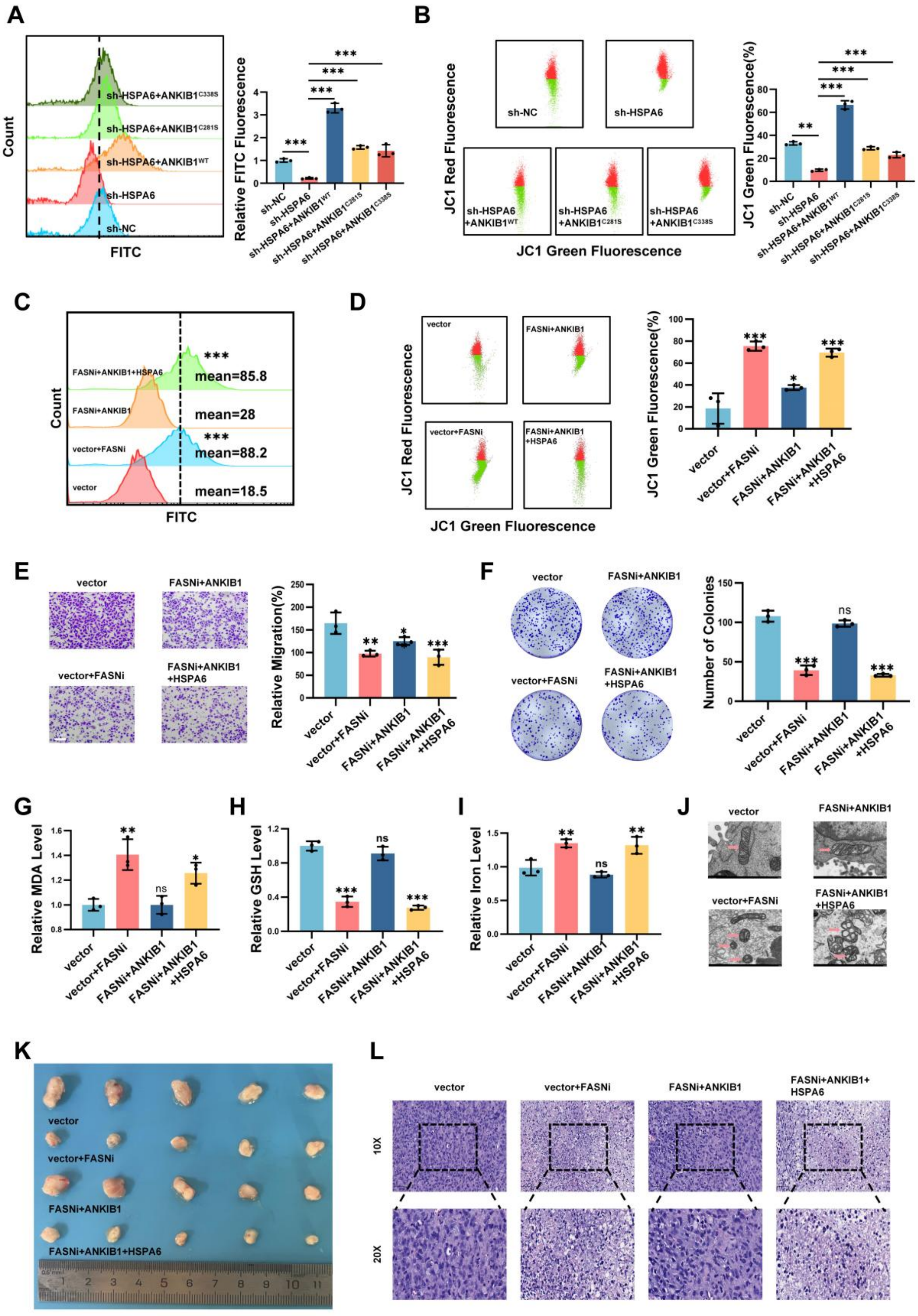
A
Count
sh-HSPA6+ANKIB1C338S
sh-HSPA6+ANKIB1C281S
sh-HSPA6+ANKIB1WT
sh-HSPA6
sh-NC
FITC
Relative FITC Fluorescence
sh-NC
sh-HSPA6
sh-HSPA6+ANKIB1WT
sh-HSPA6+ANKIB1C281S
sh-HSPA6+ANKIB1C338S
B
JC1 Red Fluorescence
sh-NC
sh-HSPA6
sh-HSPA6 +ANKIB1WT
sh-HSPA6 +ANKIB1C281S
sh-HSPA6 +ANKIB1C338S
JC1 Green Fluorescence
JC1 Green Fluorescence(%)
C
Count
FASNi+ANKIB1+HSPA6
mean=85.8
FASNi+ANKIB1
mean=28
vector+FASNi
mean=88.2
vector
mean=18.5
FITC
D
vector
FASNi+ANKIB1
vector+FASNi
FASNi+ANKIB1 +HSPA6
JC1 Red Fluorescence
JC1 Green Fluorescence
JC1 Green Fluorescence(%)
E
vector
FASNi+ANKIB1
vector+FASNi
FASNi+ANKIB1 +HSPA6
Relative Migration(%)
F
vector
FASNi+ANKIB1
vector+FASNi
FASNi+ANKIB1 +HSPA6
Number of Colonies
G
Relative MDA Level
H
Relative GSH Level
I
Relative Iron Level
J
vector
FASNi+ANKIB1
vector+FASNi
FASNi+ANKIB1 +HSPA6
K
vector
vector+FASNi
FASNi+ANKIB1
FASNi+ANKIB1+HSPA6
L
vector
vector+FASNi
FASNi+ANKIB1
FASNi+ANKIB1+ HSPA6
10X
20X

Table S1

gene	Forward Primer	Reverse Primer
β-actin	CATGTACGTTGCTATCCAGGC	CTCCTTAATGTCACGCACGAT
HSPA6	CAAGGTGCGCGTATGCTAC	GCTCATTGATGATCCGCAACAC
FASN	AAGGACCTGTCTAGGTTTGATGC	TGGCTTCATAGGTGACTTCCA
LPCAT1	ACATCCCGATCTGGGGAACT	GGCCACTTTCCGTTGGACT
LPCAT3	GGCTGGATACTATTACACTGCC	GATCTTTCCCTCCGTCAAAGTAG
	CHIP-Forward Primer	CHIP-Reverse Primer
FASN	ACAAAGGTGAGGAGATGGAGCT	TCGGAGAACTTGCAGGAGT

Table S2

gene_name	log2FoldChange	pvalue	padj
CA9	4.776067588	1.28361E-62	4.14421E-61
BEX1	3.28019549	4.96434E-26	4.26861E-25
FGF21	3.239928996	4.4526E-09	1.50197E-08
CDCA3	3.207793321	4.3405E-164	1.7094E-161
CDKN2A	2.713512705	2.43348E-56	6.50124E-55
DPEP1	2.452082681	2.80016E-63	9.26303E-62
GDF15	2.376512331	1.05383E-40	1.6493E-39
EZH2	2.312766241	2.4256E-140	5.4053E-138
CDC25A	2.169858977	8.92292E-77	4.53936E-75
CBS	1.980660485	3.41675E-23	2.57533E-22
IDO1	1.935642105	2.07262E-21	1.42043E-20
IFNG	1.865027839	4.21943E-17	2.32662E-16
CYP4F8	1.700467625	9.66235E-08	2.93396E-07
BRDT	1.685986908	1.03562E-10	3.92594E-10
HSPB1	1.63028177	2.19357E-38	3.16622E-37
FADS2	1.605755211	1.11339E-20	7.36307E-20
CHAC1	1.494221688	3.39927E-26	2.9417E-25
FANCD2	1.468998461	2.77028E-77	1.43003E-75
IDH2	1.437997078	3.80224E-57	1.03951E-55
FLT3	1.266019996	9.66451E-12	3.9305E-11
CISD3	1.225791335	7.96853E-32	8.90713E-31
GALNT14	1.206231695	2.05004E-13	9.23429E-13
ABCC5	1.145109934	1.48303E-30	1.56324E-29
ABHD12	1.102640653	2.36282E-57	6.51437E-56
CFL1	1.032325053	4.41792E-66	1.60181E-64
FZD7	-1.159058393	2.12987E-26	1.85689E-25
ACSL4	-1.178431713	3.48831E-36	4.64471E-35
CYGB	-1.330778014	2.96901E-42	4.92684E-41
CPEB1	-1.428442108	1.50914E-30	1.59003E-29
ANO6	-1.475561415	7.0466E-112	8.724E-110
DPP4	-1.493056357	1.78108E-30	1.87266E-29
AIFM2	-1.779799962	1.76358E-97	1.61135E-95
EGFR	-1.825781462	2.5849E-27	2.35659E-26
IFNA8	-1.882618297	0.001437795	0.002817978
EPAS1	-1.938304757	3.1795E-121	5.0434E-119
ACO1	-2.00635269	5.3673E-172	2.6366E-169
CREB5	-2.035931897	4.90857E-67	1.83806E-65
ATF3	-2.040186596	2.94336E-46	5.67361E-45
AKR1C3	-2.319299122	4.85403E-59	1.40438E-57
EGR1	-2.615190328	2.28686E-74	1.08713E-72
DDR2	-2.637250588	8.5437E-141	1.9322E-138
ENPP2	-2.698110746	1.26665E-100	1.22338E-98

ACSL1	-2.75286351	1.2327E-124	2.125E-122
AKR1C2	-3.00457869	1.33786E-38	1.9477E-37
CAV1	-3.360559709	2.5222E-226	7.6817E-223
AKR1C1	-3.41072927	5.76658E-69	2.29878E-67
CDO1	-3.616772231	7.8951E-120	1.2144E-117
FABP4	-4.867568213	6.8333E-101	6.64194E-99
ADIPOQ	-5.152572434	2.90123E-82	1.75317E-80
